# FzlA, an essential regulator of FtsZ filament curvature, controls constriction rate during *Caulobacter* division

**DOI:** 10.1111/mmi.13876

**Published:** 2017-12-01

**Authors:** Patrick J. Lariviere, Piotr Szwedziak, Christopher R. Mahone, Jan Löwe, Erin D. Goley

**Affiliations:** ^1^ Department of Biological Chemistry Johns Hopkins University School of Medicine Baltimore MD 21205 USA; ^2^ Structural Studies Division MRC Laboratory of Molecular Biology Cambridge CB20QH UK; ^3^ Present address: Institute of Molecular Biology and Biophysics ETH Zürich 8093 Zürich Switzerland

## Abstract

During bacterial division, polymers of the tubulin‐like GTPase FtsZ assemble at midcell to form the cytokinetic Z‐ring, which coordinates peptidoglycan (PG) remodeling and envelope constriction. Curvature of FtsZ filaments promotes membrane deformation *in vitro*, but its role in division *in vivo* remains undefined. Inside cells, FtsZ directs PG insertion at the division plane, though it is unclear how FtsZ structure and dynamics are mechanistically coupled to PG metabolism. Here we study FzlA, a division protein that stabilizes highly curved FtsZ filaments, as a tool for assessing the contribution of FtsZ filament curvature to constriction. We show that in *Caulobacter crescentus*, FzlA must bind to FtsZ for division to occur and that FzlA‐mediated FtsZ curvature is correlated with efficient division. We observed that FzlA influences constriction rate, and that this activity is associated with its ability to bind and curve FtsZ polymers. Further, we found that a slowly constricting *fzlA* mutant strain develops ‘pointy’ poles, suggesting that FzlA influences the relative contributions of radial versus longitudinal PG insertion at the septum. These findings implicate FzlA as a critical coordinator of envelope constriction through its interaction with FtsZ and suggest a functional link between FtsZ curvature and efficient constriction in *C. crescentus*.

## Introduction

Cell division in bacteria is a highly complex process that requires coordination of numerous distinct events in time and space. The cell must invaginate and remodel the inner membrane, cell wall and outer membrane, all while maintaining envelope integrity. FtsZ, an essential and widely conserved tubulin‐like GTPase (de Boer *et al*., 1992), acts as a central hub for the assembly and action of dozens of proteins (the divisome) that collectively remodel the cell envelope (Goley *et al*., 2011; Sundararajan and Goley, 2017). Prior to division, FtsZ protofilaments coalesce at midcell and, with the help of accessory proteins, form a focused superstructure called the Z‐ring (Wang and Lutkenhaus, 1996; Woldemeskel *et al*., 2017; Meier *et al*., 2017). The Z‐ring is in turn anchored to the inner membrane by adapter proteins (Ma *et al*., 1996; Pichoff and Lutkenhaus, 2005; Szwedziak *et al*., 2014; Meier *et al*., 2016). The Z‐ring successively recruits components of the divisome (Rueda *et al*., 2003; Aarsman *et al*., 2005; Goehring *et al*., 2006; Goley *et al*., 2011; Du and Lutkenhaus, 2017), which include factors involved in peptidoglycan (PG) synthesis and hydrolysis (Boyle *et al*., 1997; Weiss *et al*., 1999; Daniel *et al*., 2000; Bernhardt and De Boer, 2003; Goley *et al*., 2010a). However, the mechanisms by which the Z‐ring facilitates or organizes envelope constriction have not yet been fully elucidated.

The biochemical and biophysical properties of FtsZ are proposed to be central to its role in division (Erickson *et al*., 2010). FtsZ assembles into polymers in a guanosine nucleotide‐dependent manner, with both straight and curved filament conformations observed *in vitro* and *in vivo* (Lu *et al*., 2000; Mingorance *et al*., 2005; Li *et al*., 2007). Membrane‐targeted FtsZ is able to deform membranes in the same direction as FtsZ filament curvature (Osawa *et al*., 2009), suggesting a force generation mechanism that could be used to drive constriction (Erickson *et al*., 2010). However, it is unknown if FtsZ filament curvature contributes to division in cells or how this activity might be regulated during the tightly timed constriction process. A critical downstream outcome of Z‐ring function is synthesis and remodeling of PG at the division site, and mounting evidence suggests that this requires active direction of PG metabolism by FtsZ (Xiao and Goley, 2016; Coltharp and Xiao, 2017). In *Caulobacter crescentus*, FtsZ influences the chemical composition of the PG in a manner requiring its C‐terminal linker (Sundararajan *et al*., 2015). Moreover, recent studies in both *Escherichia coli* and *Bacillus subtilis* demonstrated that FtsZ dynamics *in vivo* drive the dynamics of PG synthetic enzymes during division (Bisson‐Filho *et al*., 2017; Yang *et al*., 2017). In *B. subtilis*, but not *E. coli*, FtsZ dynamics dictate the rate of constriction during division (Coltharp *et al*., 2016; Bisson‐Filho *et al*., 2017; Yang *et al*., 2017). It is still unknown how FtsZ and PG metabolism are mechanistically coupled or if FtsZ filament curvature plays a role in regulating PG synthesis or constriction dynamics.

Here, we have studied FzlA, a *C. crescentus* division protein that promotes formation of stable, highly curved FtsZ filaments *in vitro* (Goley *et al*., 2010b), both as a handle for probing the effect of FtsZ curvature on division, and with the aim of scrutinizing the ability of FtsZ to influence constriction rate. FzlA is an essential FtsZ‐binding protein conserved across α‐proteobacteria (Goley *et al*., 2010b). Depletion of FzlA leads to cell filamentation, but leaves Z‐ring and divisome assembly unperturbed, suggesting that FzlA is required for active constriction (Goley *et al*., 2010b; Goley *et al*., 2011). *In vitro*, FzlA not only binds to FtsZ, but promotes formation of stable, double‐stranded helices containing two highly curved FtsZ protofilaments (Goley *et al*., 2010b). We previously hypothesized that FzlA promotes constriction through its ability to regulate FtsZ filament curvature and, perhaps, the generation of FtsZ‐mediated force (Goley *et al*., 2010b).

We now present the crystal structure of FzlA and use it to inform a structure‐function analysis of FzlA with respect to its interaction with FtsZ. We demonstrate that the FtsZ‐FzlA interaction is essential for division, and that FzlA‐mediated FtsZ curvature is correlated with division efficiency. Specifically, we demonstrate that FzlA modulates the rate of constriction during division through its interaction with and curving of FtsZ filaments. We find that slowly constricting *fzlA* mutant strains form tapered, ‘pointy’ poles, consistent with our observations that mutation of *fzlA* decreases the rate of constriction relative to the rate of elongation during division. These results implicate FzlA as a key regulator of envelope constriction through its interaction with FtsZ, and are consistent with a role for FtsZ curvature in efficient cell envelope constriction in *C. crescentus*.

## Results

### FzlA forms a GST‐like homodimer

**Figure 1:**
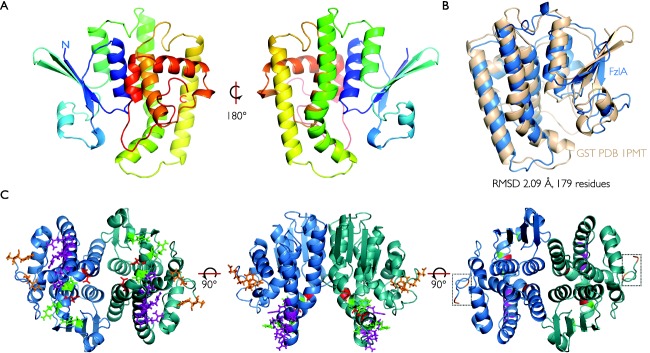
FzlA forms a homodimer in the GST structural family. A. Ribbon plot outlining the crystal structure of a FzlA monomer at 2 Å resolution. The structure is shown in rainbow colors from the N‐terminus in blue to the C‐terminus in red. B. A superposition between PDB 1PMT (RMSD of 2.09 Å over 179 Cα atoms) (cream) and FzlA (blue) is shown. C. A FzlA dimer with the relevant mutations shown: WB and UN mutations (D109R E122K and P131A) – red; UE mutations (Y223A D227K F228A) – orange; NB mutations (W38A R124D and E119K) – green; NH mutations (P131A L136A R137E, R140D E141K and R144D) – purple. The C‐termini are boxed.

To guide our investigation into the interaction of FzlA with FtsZ and its role in cytokinesis, we sought to solve its structure using X‐ray crystallography. To this end, we expressed an N‐terminal hexahistidine‐tagged version of *C. crescentus fzlA* in *E. coli*. FzlA could be purified in large quantities and crystallized readily. The cubic crystals, both native and heavy metal derivatives, diffracted well, so that it was possible to collect all the data in house (Table 1). Subsequent data processing, phasing, model building and refinement produced an electron density map at 2 Å and the corresponding model was of very good quality. It was possible to resolve residues 3–228 of FzlA and the asymmetric unit contained one molecule (Fig. 1A; Supporting Information Fig. S1 for stereo view of a portion of the structure). Based on sequence homology, FzlA was classified as a member of the GST protein family in *C. crescentus* (Goley *et al*., 2010b). Indeed, the structure of FzlA can be superimposed on that of GST from *Proteus mirabilis* (PDB 1PMT) (Rossjohn *et al*., 1998) with an RMSD of 2.09 Å over 179 Cα atoms (Fig. 1B). FzlA does not bind glutathione (Goley *et al*., 2010b), however, and lacks the catalytic histidine and serine/cysteine residues found in other GST proteins (Ma *et al*., 2009; Federici *et al*., 2010), suggesting that it no longer retains glutathione transferase activity. Applying crystal symmetry revealed that FzlA is most likely a homodimer (interface area 1263 Å^2^ as determined by PISA) (Fig. 1C). Residues 212–228 at the C‐terminus do not show any secondary structure, but are still ordered (Fig. 1C). In the course of our work, the structure of a FzlA homologue from *Sinorhizobium meliloti* (SmFzlA) was deposited in the PDB (PDB 4MDC). The two structures can be superimposed with an RMSD of 1.03 Å over 176 Cα atoms.

**Table 1:** Crystallographic data.

	FzlA native	FzlA mercury derivative
**Components**	*Caulobacter crescentus* FzlA	*Caulobacter crescentus* FzlA
GenBank IDs	ACL97219.2	ACL97219.2
UNIPROT	A0A0H3CDY2	A0A0H3CDY2
**Data collection**		
Beamline	Cu anode	Cu anode
Wavelength (Å)	1.54	1.54
**Crystal**		
Space group	I2_1_3	I2_1_3
Cell (Å)	124.33	121.77
**Scaling**		
Resolution (Å)	2.0	3.0
Completeness (%)^a^	97.3 (92.6)	99.7 (100.0)
Multiplicity^a^	4.9 (4.7)	11.9 (12.0)
Ano completeness (%)^a^		99.6 (100.0)
Ano multiplicity^a^		6.3 (6.2)
Ano correlation^[Link]^, ^[Link]^		0.285
*I*/*σI* ^a^	7.3 (1.7)	4.1 (0.5)
*R*_pim_ ^a^	0.043 (0.226)	0.081 (0.687)
CC1/2^[Link]^, ^[Link]^	0.997 (0.857)	0.996 (0.648)
**Phasing**		
Scatterer/mode		Hg
Number of sites		2
**Refinement**		
Residues	3–228	
Resolution	2 Å	
*R*‐factor, *R*‐free	0.161, 0.200	
B average^c^	28.4 Å^2^	
Geometry bonds/angles^d^	0.012 Å, 1.298°	
Ramachandran^e^	97.3%/0.0%	
**PDB ID**	**5NR1**	

**a**. Values in parentheses refer to the highest recorded resolution shell.

**b**. Correlation coefficient between half sets (CCP4 SCALA).

**c**. Temperature factors averaged for all atoms.

**d**. RMS deviations from ideal geometry for bond lengths and restraint angles.

**e**. Percentage of residues in the most and additionally favored regions of the Ramachandran plot and percentage of outliers (CCP4 PROCHECK).

Though FzlA runs as an apparent monomer by calibrated size exclusion chromatography (Goley *et al*., 2010b), both CcFzlA and SmFzlA crystallized as dimers, and we found that FzlA self‐interacts by bacterial two‐hybrid (BTH) analysis (Supporting Information Fig. S2A). As a further test for dimerization in *C. crescentus* cells, we performed co‐immunoprecipitation with differentially tagged variants of FzlA. We created a *C. crescentus* strain (EG2452) with *3xflag‐fzlA* replacing *fzlA* at its native locus and *mCherry‐fzlA* integrated at the *vanA* chromosomal locus, with expression driven by the inducible P_*van*_ promoter. Cells expressing both tagged variants of *fzlA* were lysed and anti‐FLAG conjugated agarose beads were used to immunoprecipitate 3xFLAG‐FzlA and its binding partners (Supporting Information Fig. S2B). Immunoblot analysis demonstrated that mCherry‐FzlA robustly and specifically co‐immunoprecipitates with 3xFLAG‐FzlA. Though FtsZ co‐immunoprecipitates with 3xFLAG‐FzlA in the presence of a chemical crosslinker (data not shown), it does not precipitate in the absence of crosslinker (Supporting Information Fig. S2B). These data indicate that the interaction between 3xFLAG‐FzlA and mCherry‐FzlA is direct and not mediated by FtsZ. In conjunction with the structural and BTH analysis data, these results suggest that FzlA forms a dimer *in vivo*.

### FzlA interacts with the GTPase domain of FtsZ

Since FzlA was previously shown to bind FtsZ (Goley *et al*., 2010b), we attempted to co‐crystallize FtsZ and FzlA to gain deeper insight into the nature of this interaction. Co‐crystallization proved to be unsuccessful (see Supplemental Discussion in Supporting Information), so we instead performed co‐sedimentation assays with His_6_‐FzlA and either FtsZ, FtsZΔCTL (FtsZ lacking the C‐terminal linker), or FtsZΔCTLC (FtsZ lacking the C‐terminal linker and conserved C‐terminal peptide) to determine what domain of FtsZ mediates interaction with FzlA. FzlA co‐sedimented with each FtsZ variant, suggesting that the GTPase domain of FtsZ is the site of interaction with FzlA (Supporting Information Fig. S3).

### Creation of FzlA mutant library

**Figure 2:**
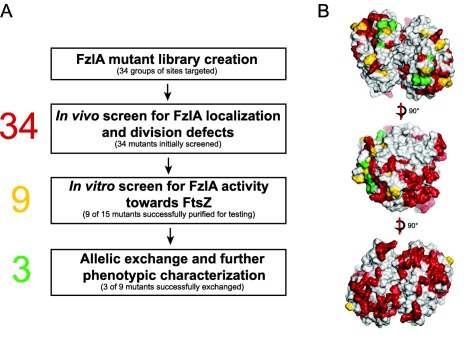
Structure‐function analysis workflow A. A list of conserved, charged and surface exposed residues were identified from the FzlA structure as potential sites of interaction with FtsZ. A library of nonconservative point mutants of 34 groups of residues was made in which the mutant proteins were fused to mCherry, with expression driven by a vanillate inducible promoter. These constructs were placed in a background where WT FzlA expression was controlled by xylose. We depleted these cells of WT FzlA and induced with vanillate, then screened for mCherry‐FzlA localization and division defects using fluorescence microscopy, growth rate analysis and spot dilution. Twenty‐five mutant genes were either associated with wild‐type looking strains or yielded protein that was unstable *in vivo* or insoluble *in vitro*, so were not further characterized. Nine mutant proteins associated with strains displaying a range of FzlA localization and division phenotypes were soluble when purified. We screened these mutant proteins for activity toward FtsZ *in vitro*, using co‐pelleting, RALS and TEM. We successfully performed allelic exchange on three of these nine mutant alleles, and further characterized their phenotypes by assessing cell length, shape, constriction rate and elongation rate. B. Dimer of FzlA showing sites originally targeted for *in vivo* screening (red), sites tested biochemically (yellow), and sites that allowed for allelic exchange (green).

With the structure of FzlA in hand, we undertook a directed mutagenesis approach to probe the role of the FzlA‐FtsZ interaction in division (Fig. 2A). We identified surface residues that were charged and/or conserved across α‐proteobacteria and used site‐directed mutagenesis to create a library of 34 *fzlA* mutant variants, each encoding a protein containing one to four nonconservative point mutations (Fig. 2B, Table 2, Supporting Information Table S1). We anticipated that these mutations might disrupt FtsZ binding, helix formation, or other unknown functions of FzlA. We were particularly interested in identifying mutations that were proficient in FtsZ binding, but deficient in curving FtsZ filaments, so that we could probe the link between FtsZ curvature and cell division.

**Table 2:** FzlA mutant phenotypes and activity.

			*P_*van*_ mChy‐mutant fzlA, P_*xyl*_ fzlA*			Mutant *fzlA* (allelic exchange)			High speed pellet	Low speed pellet		
Mutant class	Name	Residue mutations	Doubling time (min)	Max OD_600_	FzlA localization	Doubling time (min)	Max OD_600_	Cell length (µm)	Fold change of FzlA in pellet	Fold change of FzlA in pellet	% of FtsZ GTPase rate	Forms helices
WT	FzlA	n/a	131.8 ± 1.3	2.27 ± 0.0024	Midcell	108.4 ± 0.7	1.45 ± 0.03	2.71 ± 0.04	20.43 ± 1.09	11.07 ± 1.22	Trial 1: 60.63 ± 2.65 Trial 2: 48.89 ± 5.51	Y
Weakened binding to FtsZ	FzlA^WB1^	D109R E122K	135.1 ± 2.1	2.01 ± 0.0409	Diffuse	–	–	–	1.14 ± 0.13^a^	1.25 ± 0.08^a^	85.52 ± 5.32	Y
Unknown nonessential activity	FzlA^UN1^	P131A	143.4 ± 1.6	2.17 ± 0.0053	Midcell	127.3 ± 1.3	1.19 ± 0.0172	3.70 ± 0.1	9.85 ± 0.84	9.86 ± 1.56	55.69 ± 4.28	Y
Unknown essential activity	FzlA^UE1^	Y223A D227K F228A	372.4 ± 19.6	0.07 ± 0.0004	Midcell	–	–	–	5.85 ± 0.33	6.10 ± 0.55	77.33 ± 8.05	Y
	FzlA^UE2^	D227K	284.0 ± 10.0	0.19 ± 0.0206	Midcell	–	–	–	59.02 ± 2.45	12.26 ± 0.33	42.63 ± 4.28	Y
No binding to FtsZ	FzlA^NB1^	W38A R124D	142.0 ± 1.0	0.79 ± 0.0102	Diffuse	–	–	–	0.91 ± 0.14	1.25 ± 0.15	96.29 ± 6.32	N
	FzlA^NB2^	E119K	158.9 ± 0.2	0.32 ± 0.0278	Diffuse	–	–	–	1.24 ± 0.05	1.18 ± 0.16	98.90 ± 5.77	N
No helices	FzlA^NH1^	P131A L136A R137E	146.4 ± 3.0	1.00 ± 0.06	Midcell	–	–	–	2.34 ± 0.22	1.03 ± 0.02	86.25 ± 6.62	N
	FzlA^NH2^	R140D E141K	142.8 ± 2.0	2.02 ± 0.0208	Midcell	201.6 ± 2.8	0.55 ± 0.0094	4.70 ± 0.15	7.21 ± 0.18	1.30 ± 0.29	84.95 ± 5.65	N
	FzlA^NH3^	R144D	145.4 ± 2.2	2.14 ± 0.03	Midcell	132.9 ± 4.1	1.28 ± 0.0182	3.43 ± 0.07	3.51 ± 0.05	1.00 ± 0.18	89.05 ± 4.52	N

Values shown as mean ± SEM. For co‐pelleting data, values are reported as fold change of % FzlA in pellet alone vs. with FtsZ.

**a**. FzlA^WB1^ had significant pelleting on its own, making the fold change of FzlA in pellet appear low. However, this mutant still forms helices, indicating it binds FtsZ at least weakly.

As discussed below, we screened each mutant strain for FzlA localization, cell morphology and growth, and steady state FzlA protein levels *in vivo*, then selected a subset for biochemical characterization and further follow‐up (Fig. 2A). Based on our results, we created a strategy for naming the mutants for ease of reference. Eleven mutants displayed WT (W) characteristics *in vivo*, so we named these *fzlA^W1^*‐*fzlA^W11^* and did not characterize these further (Supporting Information Table S1). Seven mutants had poor protein stability (ST) by immunoblotting, named *fzlA^ST1^‐fzlA^ST7^*, and were also not pursued further. Six mutants displayed poor solubility (SOL) *in vitro*, named *fzlA^SOL1^‐fzlA^SOL6^*, which could not be purified for biochemical assays. The remaining mutants were functionally grouped and named primarily according to their biochemical activity toward FtsZ, as described below: weakened binding (WB) to FtsZ, *fzlA^WB1^*; unknown nonessential activity (UN), *fzlA^UN1^‐fzlA^UN2^*; unknown essential activity (UE), *fzlA^UE1^* & *fzlA^UE2^*; no binding to FtsZ (NB), *fzlA^NB1^*& *fzlA^NB2^*; and no helices (NH), *fzlA^NH1^‐fzlA^NH3^* (Table 2).

### fzlA mutants display a range of localization and division defects

**Figure 3:**
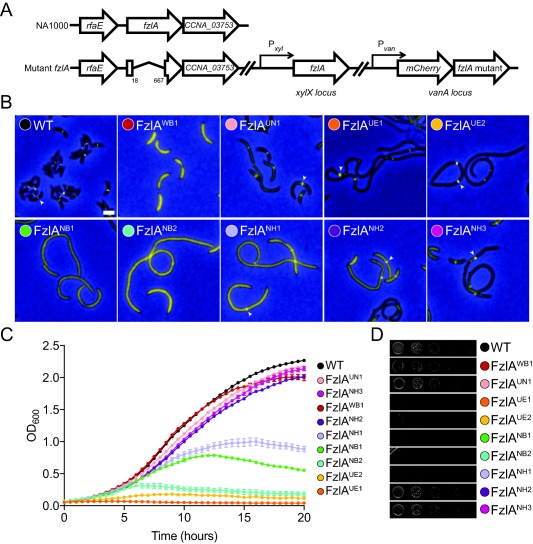
*fzlA* mutant strains display a range of division and localization deficiencies. A. Cartoon depicting genetic backgrounds for *in vivo* testing. In these strains, *fzlA* has been deleted at the native locus and a xylose‐inducible copy of *fzlA* is at the *xylX* locus. A vanillate‐inducible copy of the *mCherry‐fzlA* variant is integrated at the *vanA* locus. B. Merged fluorescence and phase contrast microscopy images depicting mCherry‐FzlA mutant protein (yellow) localization in cells depleted of WT FzlA and grown with vanillate to induce the indicated mCherry‐FzlA variant for 24 h prior to imaging. White arrowheads mark localized FzlA bands. Scale bar = 2 μm. C. Growth curves of the same strains as in (B) grown with vanillate and depleted of WT FzlA for 24 h prior to the start of the experiment. Mean of three technical replicates ± SEM is shown. D. Spot dilutions of strains as in (B), plated on PYE agar with vanillate, without predepletion of WT FzlA. Strain key: WT (EG1310), FzlA^WB1^ (EG1435), FzlA^UN1^ (EG1312), FzlA^UE1^ (EG1313), FzlA^UE2^ (EG1621), FzlA^NB1^ (EG1430), FzlA^NB2^ (EG1438), FzlA^NH1^ (EG1311), FzlA^NH2^ (EG1441), FzlA^NH3^ (EG1442).

To functionally characterize our panel of FzlA mutant strains, we devised an *in vivo* microscopy‐based assay to identify strains deficient in division and/or FzlA localization. *mCherry* was fused to the 5' end of each mutant *fzlA* allele and placed under the control of a vanillate‐inducible promoter. These fusions were integrated at the *vanA* locus in a strain background containing xylose‐inducible *fzlA* at the *xylX* locus and a *fzlA* deletion at its native locus (Fig. 3A). Cells were grown to log phase with xylose to induce WT *fzlA*, then washed and grown with vanillate to induce mutant *fzlA* and deplete WT FzlA for 24 h. Each strain was subsequently imaged by phase contrast and fluorescence microscopy and FzlA levels were probed by immunoblotting [Fig. 3B (includes mutants discussed at length); Supporting Information Figs. S4A (includes mutants that were not further characterized) and S7]. Strains were also imaged with xylose alone to only produce WT FzlA (Supporting Information Fig. S5A) or with neither inducer (Supporting Information Fig. S6A). We additionally determined growth rate and cell viability using spot dilutions to assess the fitness of each strain (Fig. 3C and D; Supporting Information Figs. S4B, S5B and S6B).

As previously shown, mCherry‐FzlA localizes to midcell and complements the loss of FzlA, yielding cells of WT length and growth rate (Goley *et al*., 2010b). Screening the library of mutant strains yielded a wide range of FzlA localization and length phenotypes in the presence of vanillate (Table 2; Supporting Information Table S1). Many of these mutants were indistinguishable from WT in viability, length and FzlA localization (*fzlA^W1^‐fzlA^W10^*) (Supporting Information Fig. S4). Numerous mutants had diffuse FzlA localization, increased cell length, and reduced growth rate and viability (*fzlA^NB1^* ‐ *fzlA^NB2^*, *fzlA^ST1^‐ fzlA^ST7^*, *fzlA^SOL1^‐ fzlA^SOL5^*) (Fig. 3; Supporting Information Fig. S4). Three mutants (*fzlA^NH1^, fzlA^UE1^, fzlA^UE2^*) had localized FzlA, yet displayed elongated cell morphologies, reductions in growth rate, and had lower colony forming units (CFUs). Five mutants (*fzlA^UN1^, fzlA^UN2^, fzlA^NH1^, fzlA^NH2^* and *fzlA^SOL6^*) similarly had FzlA localization and a mixed population of elongated and short cells, but were less impaired in growth rate and/or viability. Interestingly, one mutant (*fzlA^WB1^*) had diffuse FzlA, but was otherwise comparable to WT cells in terms of cell length, growth and viability.

It should be noted that in nearly all mutant strains (except for the poor stability mutants), vanillate induction resulted in varying degrees of mutant FzlA overexpression (Supporting Information Fig. S7). Although some of these mutant proteins appeared to be more highly expressed than others, all except the poor stability mutant proteins were expressed at levels greater than native WT FzlA. Therefore, any division defects in these mutants are unlikely to be due to insufficient protein levels. Further, the previous observation that FzlA overexpression does not result in increased cell length (Goley *et al*., 2010b), suggests mutant protein levels do not likely underlie the division defects observed *in vivo*.

Expression of WT FzlA alone in cells without induction of mCherry‐mutant FzlA supported normal growth of each strain, as expected (Supporting Information Fig. S5). Depletion of both WT and mutant FzlA protein in each strain led to cell filamentation in numerous mutant strains, but some retained WT‐like length and growth due to leaky expression from P_*van*_ (Supporting Information Figs. S6 and S8; see Supplemental Discussion in Supporting Information).

After the initial screening, we sought to visualize the Z‐ring in a subset of mutant strains deficient in division. Since depletion of FzlA does not obviously affect Z‐ring organization by conventional microscopy (Goley *et al*., 2010b), we hypothesized that mutation of FzlA would not affect it either. To test this, we imaged cells depleted of WT FzlA, producing both mCherry‐mutant FzlA and Venus‐FtsZ (Supporting Information Fig. S9). For each strain tested, we observed that Z‐ring structure was similar to WT, and that filamentous cells typically contained one or two focused Z‐rings per cell. This is similar to what has been previously reported in *C. crescentus* cells blocked for division at stages downstream of Z‐ring assembly, where relatively few Z‐rings are observed even in filamentous cells (Costa *et al*., 2008; Modell *et al*., 2014; Osorio *et al*., 2017). We also found that in strains where FzlA mutant proteins formed rings or foci, FtsZ co‐localized with FzlA. In mutant strains where FzlA was diffuse, such as *fzlA^NB1^* and *fzlA^NB2^*, FtsZ still formed rings. We, therefore, conclude that the division and/or localization defects that we observed are not due to global defects in Z‐ring assembly.

### FzlA mutant proteins display distinct defects in activities toward FtsZ in vitro

**Figure 4:**
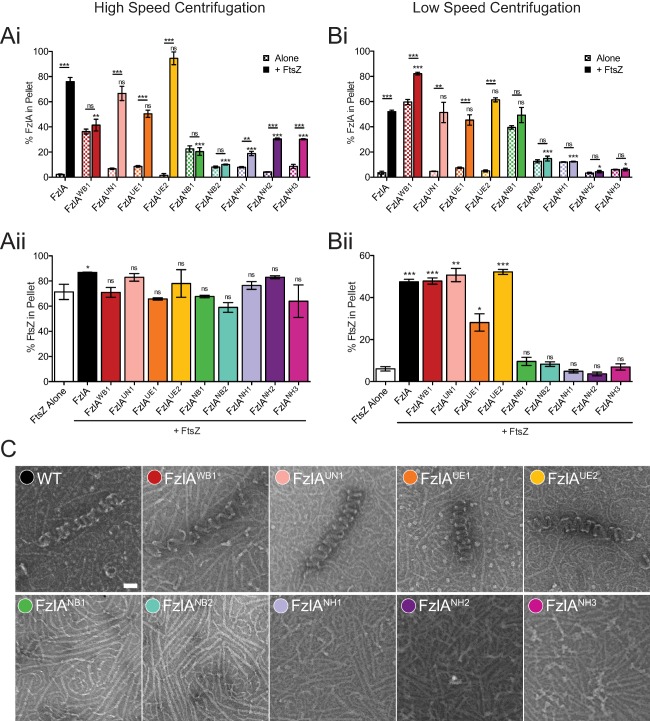
FtsZ binding and/or helix formation is perturbed for a number of FzlA mutant proteins. A, B. Quantification of the percent of WT or mutant FzlA protein (i) or FtsZ (ii) found in the pellet after high speed (A) and low speed (B) centrifugation of 4 μM FzlA variant ± 2 μM FtsZ under polymerizing conditions. Mean ± SEM is shown. For WT controls (FzlA alone, FtsZ alone and FtsZ + FzlA), representative experiments in triplicate are shown, as these controls were run numerous times. For (i), unpaired t‐tests were performed to analyze the difference between a given FzlA mutant protein alone vs. with FtsZ. Either unpaired t‐tests or a one way ANOVA with Dunnett's multiple comparison test was performed to assess differences between each FzlA mutant protein + FtsZ and WT FzlA + FtsZ (lower asterisks), depending on the number of mutants in a particular run. For (ii), a one way ANOVA with Dunnett's multiple comparison test was performed to assess differences between WT FzlA or mutant FzlA + FtsZ vs. FtsZ alone. For all statistical tests, ^ns^ *P* > 0.05, ^*^ *P* ≤ 0.05, ^**^ *P* ≤ 0.01, ^***^ *P* ≤ 0.001 and *n* = 3 for each sample. For the Commassie‐blue SDS‐PAGE gels from which pelleting data is drawn, see the Supporting Information. C. Negative stain TEM images of 4 μM FzlA variant + 2 μM FtsZ under polymerizing conditions. Scale bar = 50 nm.

Having screened the *fzlA* mutant strains for localization and division phenotypes *in vivo*, we compiled a list of mutant proteins for characterization of activity toward FtsZ *in vitro*. We did not pursue the *fzlA^W^* or *fzlA^ST^* mutants, since they were fully functional or displayed poor protein stability *in vivo* by immunoblot analysis respectively (Supporting Information Fig. S7). We additionally did not follow up on FzlA^UN2^, since its mutated residue (Y223A) was included in another mutant, FzlA^UE1^ (Y223A, D227K, F228A) and the phenotype of the triple mutant was identical to that of FzlA^UE2^ bearing the single D227K mutation. We attempted to purify the remaining 15 mutant proteins by expressing them as His_6_‐SUMO fusions, followed by affinity purification and cleavage of the His_6_‐SUMO tag. Six of these mutant proteins (FzlA^SOL1^‐FzlA^SOL6^, Supporting Information Table S1) were insoluble when expressed in *E. coli*, so we focused our efforts on the nine biochemically tractable mutant proteins that corresponded with non‐WT phenotypes *in vivo* (Table 2). Following purification, we subjected these nine mutant proteins to a series of biochemical assays, testing for FtsZ binding, higher order structure formation and helix formation (Fig. 4). We first optimized these assays using untagged WT FzlA, since the conditions previously reported used His_6_‐FzlA (Goley *et al*., 2010b) (see Supplemental Discussion in Supporting Information). In the following experiments, we used the *in vitro* conditions that maximized WT FzlA activity (pH 6.5, 50 mM KCl), allowing us to probe the activities of different mutant proteins and identify even subtle defects. These conditions are in line with commonly used conditions for FtsZ activity assays (Chen *et al*., 2007; Pacheco‐Gómez *et al*., 2011; Milam and Erickson, 2013).

To test for binding to FtsZ, we performed a high speed co‐pelleting assay in which FtsZ polymers pellet on their own and can bring bound FzlA to the pellet (Fig. 4A; Supporting Information Fig. S10). In the absence of FtsZ polymers, FzlA remains in the supernatant (Goley *et al*., 2010b) (Fig. 4Ai). To quantify formation of higher order structures, we used a low speed co‐pelleting assay where neither FzlA nor FtsZ polymers pellet in isolation, but both co‐sediment when combined under polymerizing conditions due to formation of large helical structures (Goley *et al*., 2010b) (Fig. 4B; Supporting Information Fig. S11). To confirm the formation of higher order structures, we used right‐angle light scattering (RALS) (Supporting Information Fig. S12). Lastly, we used negative stain transmission electron microscopy (TEM) to directly observe helix formation.

It has been previously shown that WT FzlA co‐pellets with FtsZ at both high and low speeds, forms large structures by RALS, and makes helices by TEM (Goley *et al*., 2010b). Subjecting the nine biochemically tractable mutant proteins to these assays gave a range of activities compared to WT. FzlA^UN1^ (UN = unknown nonessential) behaved similarly to WT FzlA in all assays (Fig. 4). Two additional mutant proteins (FzlA^UE1^ & FzlA^UE2^; UE = unknown essential activity) also bound to FtsZ and formed helices, but were binned into a separate group based on their phenotypes *in vivo* (discussed below). FzlA^NB2^ (NB = no binding to FtsZ) was found to neither bind nor form helices or other higher order structures with FtsZ. Additionally, three mutant proteins (FzlA^NH1^, FzlA^NH2^, FzlA^NH3^; NH = no helices) appeared to uncouple FtsZ binding from helix formation: they bound to FtsZ, albeit to a lesser extent than WT FzlA, but did not form helices.

Two mutant proteins, FzlA^WB1^ (WB = weak binding to FtsZ) and FzlA^NB1^(NB = no binding to FtsZ), appeared to self‐aggregate (Fig. 4Ai), making it difficult to assess binding to FtsZ by high speed co‐pelleting under the standard conditions used in this study (pH 6.5, 2:1 FzlA:FtsZ). Increasing pH to 7.2 and using a 1:1 FzlA:FtsZ ratio significantly reduced aggregation and allowed us more confidently assess binding by high speed pelleting. Under these conditions, neither mutant co‐sedimented with FtsZ significantly better than when alone (Supporting Information Fig. S13). Interestingly, under our standard conditions, FzlA^WB1^ still formed structures by low speed pelleting and helices by TEM, whereas FzlA^NB1^ did not (Fig. 4B and C). FzlA^WB1^, therefore, appears to weakly bind FtsZ since it is able to form helices, while FzlA^NB1^ does not bind at all.

FzlA was previously shown to reduce FtsZ's GTPase activity (Goley *et al*., 2010b), similar to other FtsZ polymer stabilizing proteins (Gueiros‐Filho and Losick, 2002; Hale *et al*., 2011; Durand‐Heredia *et al*., 2012). We measured GTPase rate of FtsZ in the presence of FzlA mutant proteins to determine if variants retained the ability to lower activity (Table 2). The mutant proteins that interacted more strongly with FzlA and formed helices lowered the GTPase rate more than the weak interactors, though FzlA^WB1^ (which forms helices, but weakly binds FtsZ) did not significantly lower FtsZ's GTPase rate. From these data we conclude that reduction in GTPase rate generally correlates with helical FzlA‐FtsZ bundle formation.

### FzlA‐FtsZ binding is necessary for allelic complementation

**Figure 5:**
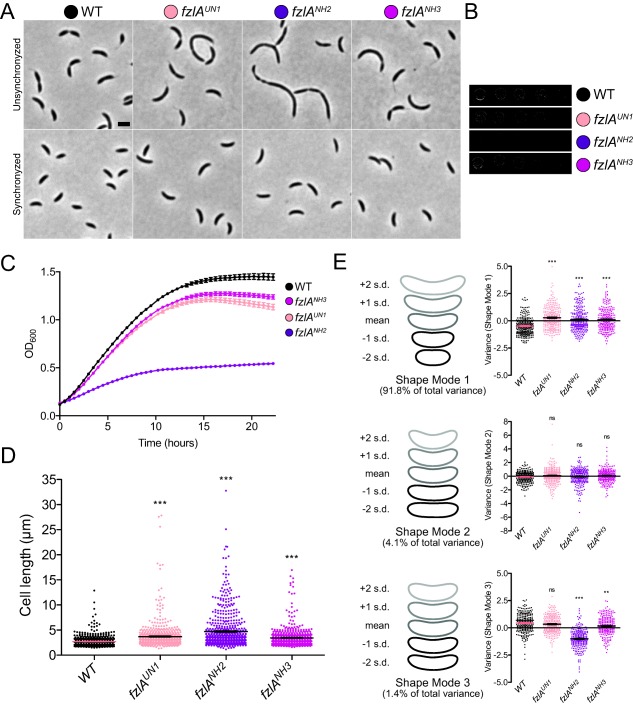
Three *fzlA* mutant genes are capable of allelic replacement, causing a range of division and morphological defects. A. Phase contrast images of unsynchronized (top) and synchronized (bottom) cells from strains carrying mutant *fzlA* alleles as the only copy. Scale bar = 2 μm. B. Spot dilutions of mutant *fzlA* allelic exchange strains. C. Growth curves of mutant *fzlA* allelic exchange strains. Mean of three technical replicates ± SEM is shown. D. Lengths of unsynchronized cells from *fzlA* allelic exchange strains. Mean ± SEM is shown. A one‐way ANOVA with a Kruskal‐Wallis test was performed to analyze differences compared to WT: ^***^ *P* ≤ 0.001. From left to right, *n* = 610, 618, 607, 610. E. PCA of cell shape in synchronized *fzlA* allelic exchange strains. For each of the three shape modes that account for most of the variance in the population, mean cell contour ± 1 or 2 standard deviations (s.d.) is shown (left). Shape mode values for cells in each strain are plotted and mean ± SEM is indicated (right). A one‐way ANOVA with with a Kruskal‐Wallis test was performed to assess differences compared to WT: ^ns^ *P* > 0.05, ^**^ *P* ≤ 0.01, ^***^ *P* ≤ 0.001. From left to right, *n* = 261, 288, 249, 245. Strain key: WT (NA1000), *fzlA^UN1^* (EG1908), *fzlA^NH2^* (EG1600), *fzlA^NH3^* (EG1909).

To clarify the contributions of FzlA's biochemical activities to cell division, we next asked if any of the nine *fzlA* mutant alleles corresponding with the mutant proteins we characterized biochemically were able to replace WT *fzlA* as the only copy of the gene in the cell. To this end, we attempted allelic exchange at the *fzlA* locus using each of the above nine mutant genes. However, we were only able to successfully replace WT *fzlA* with three of these (*fzlA^UN1^, fzlA^NH2^, fzlA^NH3^*) (Fig. 5A; Supporting Information Fig. S14). The proteins corresponding to each of these three mutant alleles bound to FtsZ to some extent and, conversely, the alleles corresponding to binding‐deficient mutant proteins (*fzlA^NB1^* & *fzlA^NB2^*) were unable to complement loss of *fzlA*. These observations indicate that the FzlA‐FtsZ interaction is essential for viability and division. Additionally, the alleles corresponding to the proteins that bound FtsZ and formed helices *in vitro*, but themselves caused cell elongation and very severe growth defects (*fzlA^UE1^* & *fzlA^UE2^) in vivo*, also could not complement loss of *fzlA*. Since the residues altered in the proteins corresponding with these two mutant alleles do not appear to be necessary for interaction with FtsZ *in vitro*, we propose that they mediate an unknown, but essential activity distinct from FtsZ binding. Interestingly, although *fzlA^WB1^* cells were not particularly sick in the FzlA depletion experiment (Fig. 3), *fzlA^WB1^* could not replace WT *fzlA* on the chromosome. This was somewhat surprising, but we reason that since induction with vanillate causes overexpression of the mutant allele (Supporting Information Fig. S7), higher protein levels may compensate for FzlA^WB1^'s weak affinity for FtsZ.

### Mutant fzlA strains display growth and shape defects

We characterized the phenotypes of the *fzlA* allelic exchange strains in detail to further assess the link between *in vitro* activity of FzlA and its *in vivo* function. *fzlA^UN1^* and *fzlA^NH3^* grew close to WT by spot dilution (Fig. 5B), but displayed a moderate reduction in growth rate (Fig. 5C) and were elongated (Fig. 5A and D). The phenotype of *fzlA^NH2^* was much more severe, as evidenced by reduced colony size and number (Fig. 5B), significantly lower growth rate (Fig. 5C), and elongated and filamentous cells (Fig. 5A and D). The steady state protein levels of FzlA^UN1^ and FzlA^NH3^ were similar to WT, while there was a moderate increase in levels of FzlA^NH2^ (Supporting Information Fig. S14). The mild overexpression of *fzlA^NH2^* is unlikely to underlie the morphological defects observed, however. As previously mentioned, overexpression of *fzlA* (Goley *et al*., 2010b), *mCherry‐fzlA*, or many of the *mCherry‐fzlA* mutant variants we tested (Supporting Information Fig. S7), did not result in significant cell length defects.

We sought to quantify cell shape abnormalities of each mutant strain using Celltool (Pincus and Theriot, 2007). To this end, we synchronized mutant and WT cells to minimize cell cycle‐dependent differences in shape and imaged them prior to the initiation of constriction. Cell contours from WT and the three mutant strains were extracted and principle component analysis (PCA) was performed to determine variation in shape across the four populations. The modes which accounted for the most variation from mean cell shape roughly reflected cell length, curvature and width (Fig. 5E). Each mutant strain was elongated compared to WT, consistent with our cell length measurements of a mixed population of cells. In addition, *fzlA^NH2^* cells and to a lesser extent, *fzlA^NH3^* cells, were skinnier than WT. Cell curvature did not appear to be affected by mutations in *fzlA*. In sum, the three *fzlA* strains capable of allelic exchange were distinct from WT in growth rate, cell length and cell width, with *fzlA^NH2^* having the most severe phenotype.

Characterizing the phenotypes of the allelic exchange strains allowed us to more precisely correlate the biochemical activity of their corresponding mutant proteins with their division defects. While FzlA^NH1^, FzlA^NH2^ and FzlA^NH3^ were found to bind FtsZ, they do not form helices or higher order structures (Fig. 4A–C). As shown above, expression of each of these mutant genes in a FzlA depletion background (*fzlA^NH1^*) or on its own (*fzlA^NH2^, fzlA^NH3^*) led to division defects. *fzlA^NH2^* and *fzlA^NH3^* had severe and mild deficiencies respectively, in growth rate, length and shape. *fzlA^NH1^*, conversely, was not capable of allelic replacement. From this we conclude that, while FzlA‐FtsZ helices are not required for division, loss of these structures *in vitro* does correlate with a decrease in division efficiency. The mechanisms behind the mild growth phenotypes observed in *fzlA^UN1^*, which still forms helices, are not currently clear. We observed a slight, but not statistically significant, reduction in FzlA^UN1^ co‐pelleting, which might reflect mildly reduced binding affinity for FtsZ (Fig. 4Ai).

### fzlA mutant strains display reduced constriction rates

**Figure 6:**
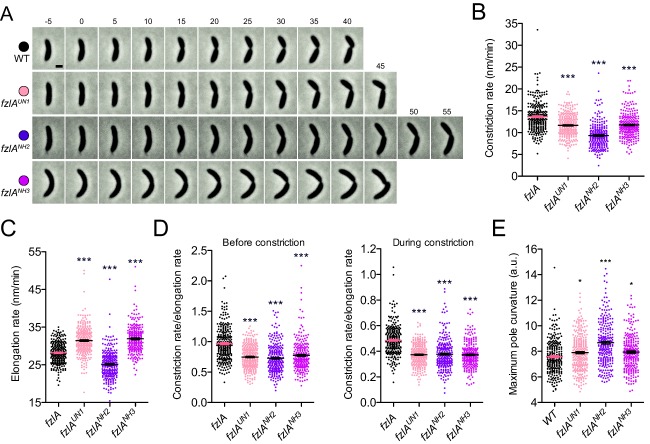
*fzlA* mutant strains show protracted constriction. A. Phase contrast images of representative constricting cells from *fzlA* allelic exchange strains, acquired at 5‐min intervals. For each strain, constriction begins in the second frame (*t* = 0) and ends on cell separation in the last frame. Scale bar = 1 μm. B, C. Plots of constriction rate (B) and elongation rate during constriction (C) for a population of synchronized cells from each *fzlA* strain, calculated from single cell microscopy data. Mean ± SEM is shown. One‐way ANOVA tests with Tukey's multiple comparison test were performed to analyze differences compared to WT: ^***^ *P* ≤ 0.001. From left to right, *n* = 271, 295, 260, 249 (B) and 266, 293, 258, 246 (C). D. Plots of the ratio of constriction rate to elongation rate before constriction (left) and during constriction (right) for a population of synchronized cells from each *fzlA* strain, calculated from single cell microscopy data. Mean ± SEM is shown. One way ANOVA tests with Tukey's multiple comparison test were performed to analyze differences compared to WT: ^***^ *P* ≤ 0.001. From left to right, *n* = 271, 295, 260, 249 (left) and 266, 293, 258, 246 (right). E. Plot of the maximum curvature of the poles for a synchronized population of single cells for each *fzlA* allelic exchange strain. Mean ± SEM is shown. A one‐way ANOVA with a Kruskal‐Wallis test was performed to analyze differences compared to WT: ^*^ *P* ≤ 0.05, ^***^ *P* ≤ 0.001. From left to right, *n* = 261, 288, 249, 245. Strain key: WT (NA1000), *fzlA^UN1^* (EG1908), *fzlA^NH2^* (EG1600), *fzlA^NH3^* (EG1909).

Mutation of fzlA has a clear effect on the efficiency of division. Additionally, we previously showed that depletion of FzlA leads cells to grow into smooth filaments without affecting divisome assembly, indicating a role in the constriction process itself (Goley *et al*., 2010b). We, therefore, hypothesized that FzlA regulates the initiation and/or rate of constriction and that this rate may be impaired in the *fzlA* mutant strains. To address this possibility, we performed single‐cell timelapse microscopy on WT, *fzlA^UN1^, fzlA^NH2^* and *fzlA^NH3^* cells. Cells from each strain were synchronized, then placed onto an agarose pad for imaging by phase‐contrast microscopy at five minute intervals (Fig. 6A; Supporting Information Video S1–S4). MicrobeJ (Ducret *et al*., 2016) was used to track individual cells, quantify cell length and width at each time point, and mark time of constriction initiation. The time of completion of division was manually scored for each cell. From these data, the number of frames required to complete constriction was determined for single cells, giving the constriction time. We then calculated constriction rate by dividing the change in cell width during constriction by constriction time (Fig. 6B). Strikingly, the constriction rates for *fzlA^UN1^* (11.7 ± 0.1 nm/min) and *fzlA^NH3^* (11.7 ± 0.2 nm/min) were decreased compared to WT (13.6 ± 0.2 nm/min). Consistent with the more severe phenotype of *fzlA^NH2^*, these cells constricted even more slowly (9.3 ± 0.2 nm/min).

The decrease in constriction rates observed for the *fzlA* mutant strains might be explained by an overall decrease in the rate of cell wall synthesis. If this was the case, we would expect to see a corresponding decrease in elongation rate, which also depends on cell wall synthesis, for each strain. Elongation rate during constriction was actually increased by a small degree for *fzlA^UN1^* (31.4 ±0.2 nm/min) and *fzlA^NH3^* (31.9 ± 0.2 nm/min), compared to WT (28.2 ± 0.2 nm/min), whereas it was slightly decreased for *fzlA^NH2^* (25.2 ± 0.2 nm/min) (Fig. 6C). We, therefore, examined the ratio of constriction rate to elongation rate for cells of each strain to account for possible global differences in cell wall synthesis (Fig 6D). Importantly, we found that this ratio decreased by 20%–25% in each mutant strain compared to WT. We observed similar trends when we compared constriction rate to the elongation rate prior to constriction or to the elongation rate during constriction. We, therefore, conclude that the decrease in constriction rate of each mutant is not merely due to a decrease in global cell wall production, but rather includes a constriction‐specific defect.

We additionally observed an increase in the time of constriction initiation (preconstriction time) in *fzlA^NH2^* cells, but not for *fzlA^UN1^* or *fzlA^NH3^* (Supporting Information Fig. S15A), indicating that this mutation in *fzlA* results in delayed constriction onset. The change in constriction timing is correlated with a slight increase in the change in cell length before constriction (Supporting Information Fig. S15B). Finally, we found in all of the *fzlA* allelic exchange mutants that the decreased constriction rates (Fig. 6D) and increased constriction times are correlated with greater changes in length during constriction (Supporting Information Fig. S15C, D), as expected.

To further validate the observed differences in constriction versus elongation rates for the *fzlA* mutants, we assessed cell pole shape. Pole shape is indicative of the relative rates of constriction and elongation: a mutant strain that constricts faster (without a corresponding increase in elongation rate) forms blunter poles, while one that constricts slower (without a corresponding decrease in elongation rate) forms more tapered, pointed poles. We, therefore, determined the maximum instantaneous curvature at the cell poles as a readout for pole ‘pointiness’ (Fig. 6E). *fzlA^NH2^* cells, and to a lesser degree *fzlA^UN1^* and *fzlA^NH3^* cells, were found to have significantly ‘pointier’ poles than WT. These data are consistent with our observation that while the constriction rate (i.e., rate of decrease of the diameter of the cell) had been lowered, the elongation rate (i.e., rate of longitudinal insertion of cell wall material) was not affected to the same degree. We conclude that FzlA, through its interaction with FtsZ, plays a specific role in regulating the rate of constriction during *C. crescentus* division.

## Discussion

**Figure 7:**
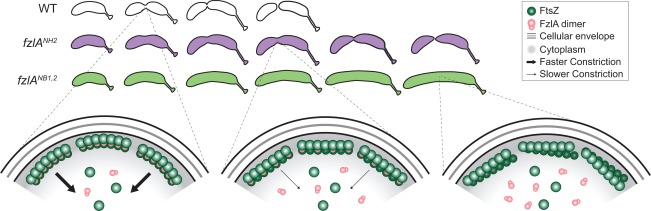
Model of FzlA function in regulating constriction. Full interaction with FtsZ, including helix formation, is required for wild‐type constriction rate. Top: constriction is shown for normally dividing WT cells (white) and slower dividing *fzlA^NH2^* cells (purple). *fzlA^NB1,2^* cells (bright green) grow, but do not constrict or divide. Bottom: Close‐up of the division septa, depicting FtsZ (dark green) bound to WT or mutant FzlA protein (pink). WT FzlA binds and curves FtsZ *in vitro*, and corresponds with normal, fast constriction (bottom left). FzlA^NH2^ binds, but does not curve FtsZ filaments, *in vitro* and correlates with slower constriction (bottom middle). (Note, FzlA^NH2^ is shown to bundle FtsZ filaments, however, it is unclear by TEM if this is indeed the case.) FzlA^NB1–2^ mutant proteins do not bind or curve FtsZ *in vitro*, which prohibits constriction and division (bottom right).

In this study, we undertook a structure‐function approach to characterize the role of FzlA in division in *C. crescentus*. We solved the structure of FzlA and found that it forms a GST‐like dimer, then created a library of point mutants with the goal of altering its interaction with FtsZ. After correlating *in vivo* localization and function with *in vitro* activity toward FtsZ for each mutant allele and corresponding protein, we demonstrated that FzlA binding to FtsZ is required for division (Fig. 7). Further, the ability of FzlA to curve FtsZ filaments, while not essential, is correlated with the efficiency of division (Fig. 7). Importantly, FzlA appears to play a key role in determining cell constriction rate through its interaction with FtsZ (Fig. 7).

Our structural, BTH analysis, and co‐immunoprecipitation data provide evidence that FzlA forms a dimer. In the co‐immunoprecipitation, 3xFLAG‐FzlA is able to pull down a roughly equimolar amount of mCherry‐FzlA and other FzlA species that are not reactive with the FLAG antibody (e.g., mCherry‐FzlA and 3xFLAG‐FzlA degradation products), suggesting the interaction is unlikely to be the result of binding to a bridging protein (Supporting Information Fig. S2B). That FzlA dimerizes potentially provides insight into how FzlA is able to mold FtsZ protofilaments into double stranded helices. The most straightforward model is that monomers within a FzlA dimer may bind to FtsZ subunits on opposing strands, positioning them in an offset orientation that causes twisting and helix formation on polymerization. Alternatively, it is possible that each monomer within a FzlA dimer binds to discrete FtsZ subunits on a single strand, imposing a slight twist. Each strand may then bind to a second either through intrinsic lateral interactions or potentially via self‐association of FzlA dimers. In any case, since we never observed highly curved single FtsZ filaments in the presence of WT or any mutant FzlA protein, we propose that FzlA's curvature‐inducing activity requires and/or is linked to pairing of FtsZ filaments.

FtsZ has been shown to exist in a curved conformation *in vivo* (Li *et al*., 2007), but little is known about the coordination and specific function of curved conformations of FtsZ. So far, only a few proteins have been implicated in FtsZ curvature regulation: *B. subtilis* ZapA forms mini‐rings with FtsZ in the absence of nucleotide (Gueiros‐Filho and Losick, 2002), *Thermotoga maritima* FtsA curves FtsZ filaments on membranes through the repeat distance mismatch (Szwedziak *et al*., 2012; Szwedziak *et al*., 2014; Ghosal and Löwe, 2015), and FzlA curves FtsZ through the formation of double‐stranded helices (Goley *et al*., 2010b). In the current study, we focused on the FtsZ‐FzlA interaction and have specifically identified sites that contribute to binding to FtsZ. Mutation of one set of residues (D109 and E122 [FzlA^WB1^]) weakens interaction with FtsZ, while perturbation of others (W38, R124 [FzlA^NB1^] and E119 [FzlA^NB2^]) completely eliminates binding. In addition, we identified residues (P131, L136, R137 [FzlA^NH1^], R140, E141 [FzlA^NH2^] and R144 [FzlA^NH3^]) that when mutated, retained some ability to bind but were no longer able to curve FtsZ filaments. Satisfyingly, these residues map to the same face on FzlA's surface (Fig. 1C). The residues required for curvature all border one another and while the residues required for binding are nearby on the same face, they are not directly adjacent to each other. This region of FzlA is likely in physical contact with FtsZ, though not all of these residues necessarily directly interact with FtsZ.

Though our mutational analysis suggests a link between FzlA‐FtsZ helices and division efficiency, we surprisingly found that the ability of FzlA to interact with FtsZ and promote formation of helices is not sufficient for division. Two mutant proteins (FzlA^UE1^ & FzlA^UE2^) localize *in vivo*, robustly bind to FtsZ, and form helices, yet their corresponding strains are unable to function in division. The altered residues in these mutant proteins (Y223, D227 and F228) are located close to the C‐terminus of FzlA, a region that is spatially distant from the FtsZ binding residues on the surface of FzlA; this separation likely explains why their mutation had little impact on interaction with FtsZ (Fig. 1C). The Y223A (*fzlA^UN2^*) and F228A (*fzlA^W11^*) single mutant strains were viable, while the D227K (*fzlA^UE2^*) mutant strain was indistinguishable from the triple mutant strain (*fzlA^UE1^*), suggesting that mutation of D227 (*fzlA^UE2^*) is largely responsible for the severe division defect associated with mutation of the C‐terminus (Supporting Information Fig. S4; Fig. 3). Interestingly, the C‐terminus of FzlA is a highly acidic region of about 20 amino acids lacking secondary structure. While some residues in this region, including F228A, make contacts into the hydrophobic core of the protein, the functionally critical D227 residue is exposed, making it a possible protein interaction site. The C‐terminus may regulate FzlA's interaction with itself, though we did not observe an interaction of the isolated C‐terminus with FzlA by BTH analysis (data not shown). Alternatively, the C‐terminal tail may bind to an unidentified, essential division protein, possibly through residue D227.

Previous work has correlated FtsZ curvature with membrane deformation *in vitro*, suggesting a role in force generation (Osawa *et al*., 2009). Though other studies indicate FtsZ polymers can occupy a curved conformation *in vivo* (Lu *et al*., 2000; Li *et al*., 2007; Li *et al*., 2013), FtsZ curvature has not previously been linked to an effect on division in cells. Through our combination of *in vivo* phenotypic characterization and biochemical assays, we describe a mutant protein (FzlA^NH2^) that binds FtsZ, but is incapable of forming helices, and that is associated with severe growth, division and shape defects. Interestingly, loss of helix formation yielded a range of phenotypes in other mutant strains, from a milder effect on division and constriction rate (*fzlA^NH3^*), to an inability to complement loss of *fzlA* (*fzlA^NH1^*). We conclude that, though not strictly essential, FzlA‐induced FtsZ curvature is correlated with efficient division. Based on previously described models (Erickson *et al*., 2010), it is possible that FzlA‐mediated FtsZ curvature directly enables constriction through contractile force generation.

It is important to note that the structure of the FzlA‐FtsZ complex *in vivo* is currently unknown. That FzlA and FtsZ form helices *in vitro* does not necessarily mean they form these structures *in vivo*. Indeed, highly curved or helical FtsZ polymers have not been observed by cryo‐electron tomography (cryo‐ET) in *C. crescentus* cells (Li *et al*., 2007; Szwedziak *et al*., 2014; Yao *et al*., 2017). However, it is possible that FzlA and FtsZ do form highly curved structures *in vivo* that have not yet been resolved by cryo‐ET since we lack methods for specifically labeling proteins for cryo‐ET imaging. Alternatively, or in addition, the highly curved helices observed *in vitro* by TEM may be in a relaxed or unconstrained state; in cells, they may be constrained through membrane attachment to appear less curved [i.e., to follow the curvature of the cell membrane, much like is proposed for crescentin (Cabeen *et al*., 2009)]. Finally, it is possible that FzlA does not stabilize a highly curved form of FtsZ in cells, and that helix formation simply occurs under some conditions *in vitro* with full, WT binding of FzlA to FtsZ. Although demonstration of helix formation by TEM does not necessarily indicate helix formation in cells, the properties of FzlA that allow it to fully interact with FtsZ and promote helix formation *in vitro* are clearly important for its role in promoting constriction in cells.

Our observations also indicate that the interaction of FzlA with FtsZ filaments influences constriction rate, which logically would be mediated through peptidoglycan (PG) metabolism. Recent work has shown that FtsZ dynamics regulate PG synthesis, and that slowing FtsZ turnover by reducing its GTPase rate causes cell envelope morphology defects in *E. coli* and slowed constriction rate in *B. subtilis* (Bisson‐Filho *et al*., 2017; Yang *et al*., 2017). Our study provides evidence that FtsZ regulates constriction rate in *C. crescentus*, but also suggests that FtsZ polymer structure may be important for this regulation. Interestingly, while mutation of FzlA lowered the constriction rate (68.4% of WT constriction rate), the cell elongation rate was not altered to the same degree (89.3% of WT elongation rate) (Fig. 6 **C**). Partial inactivation of the septal PG transpeptidase FtsI in *E. coli* also leads to a decrease in constriction rate compared to elongation rate (Coltharp *et al*., 2016) and to formation of pointed cell poles (Taschner *et al*., 1988; Costa *et al*., 2008). These data not only confirm that mutation of FzlA has a greater impact on constriction rate over elongation rate, but also suggest a potential genetic link between FzlA and the PG synthetic enzyme, FtsI.

The observation that the cell poles become more ‘pointy’ in the *fzlA^NH2^* mutant strain indicates that new cell wall material is still being added at the division site, but with a slower decrease in cell diameter than for WT. We can envision at least two possible mechanisms underlying this apparent uncoupling of PG synthesis rate (i.e., elongation rate) and constriction rate. In the first, FzlA influences the enzymatic activity of cell wall enzymes specific to division (e.g., FtsW and FtsI), and when FzlA is mutated to slow the constriction rate, the total activity of these enzymes is reduced. In this case, much of the new material being inserted at midcell must be mediated by the elongasome (e.g., RodA and PBP2) or general PG synthetic enzymes (e.g., the bifunctional PBP family) to maintain the elongation rate. Alternatively, all of the new material being inserted at midcell is synthesized by the divisome in the *fzlA^NH2^* mutant strain. In this case, the directionality of insertion in a radial direction – but not overall enzymatic activity of PG enzymes in the divisome – requires input from FzlA and FtsZ.

The role of FtsZ in defining the rate of division, as well as the contribution of FtsZ curvature to division, has been longstanding questions in the field. Through mutational analysis, we provide evidence in this study that a regulator of FtsZ curvature, FzlA, affects the rate of constriction for *C. crescentus* cytokinesis. Future work will be aimed at determining if the FzlA‐FtsZ interaction facilitates division through force generation, by communication with PG remodeling enzymes, or a combination of the two.

## Experimental procedures

### Bacterial strains, plasmids and growth conditions

*C. crescentus* NA1000 strains were grown at 30°C in peptone yeast extract (PYE) medium, as previously described (Meier *et al*., 2016). Inducers and antibiotics were added to liquid (and solid) PYE media at the following concentrations: 0.3 (0.3)% xylose, 0.3 (0.3)% glucose, 0.5 (0.5) mM vanillate, 5 (25) µg ml^−1^ kanamycin, 25 (100) µg ml^−1^ spectinomycin, (5 µg ml^−1^) streptomycin and 1 (1) µg ml^−1^ chloramphenicol. Prior to induction or depletion, cells were washed three times in plain PYE. Growth rates were determined using a Tecan Infinite 200 Pro plate reader, with strains grown in a 96‐well plate shaking at 30°C. Spot dilutions were performed by first growing cells in liquid culture to OD = 0.1, serially diluting by 10^−1^ to 10^−7^, then plating 5 µl of the 10^−2^ to 10^−7^ dilutions. Cells were synchronized where indicated, as previously described (Goley *et al*., 2011). Strains and plasmids used are detailed in the Supporting Information (Supporting Information Tables S2 and S3).

### Protein purification

FtsZ, FtsZΔCTL and FtsZΔCTLC were expressed in *E. coli* Rosetta (DE3) pLysS and purified as previously described (Sundararajan *et al*., 2015). To purify His_6_‐FzlA for crystallization and co‐sedimentation, FzlA (GenBank ID ACL97219.2, UNIPROT number A0A0H3CDY2) was cloned with an N‐terminal His_6_‐tag. The protein was expressed at 37°C in *E. coli* C41 (DE3) cells for 4 h. Cells were lysed in buffer A (50 mM Tris, 300 mM NaCl, 40 mM imidazole, pH 8.0) by passing through a Constant Systems cell disruptor at 25 kpsi. The soluble fraction was loaded on a nickel column (2 × 5 ml HisTrap, GE Healthcare), washed thoroughly with 250 ml buffer A and the protein was eluted by 0–1 M imidazole gradient. Peak fractions were pooled, concentrated and subjected to size‐exclusion chromatography on Sephacryl S200 (GE Healthcare) in buffer B (50 mM Tris, 300 mM NaCl, pH 8.0). Peak fractions were concentrated to 10 mg/ml and stored at −80°C.

To purify untagged FzlA for biochemical characterization, His_6_‐SUMO‐FzlA or FzlA mutant was first expressed at 30°C in Rosetta (DE3) pLysS *E. coli* cells for 4 h following induction with 0.25–1 mM isopropylthioglactoside (IPTG). Cells were pelleted by centrifugation at 6000 × g for 10 min at 4°C, then re‐suspended in 40 ml buffer A (50 mM HEPES‐KOH, 300 mM KCl, 20 mM imidazole, 10% glycerol, pH 7.2) per 1 l of culture and snap frozen in liquid nitrogen. The cell suspension was thawed and incubated at room temperature for 1 h, after receiving the following additives: 1 mg ml^−1^ lysozyme, 2 mM phenylmethyl sulphonyl fluoride (PMSF), 2 units ml^−1^ DNAse I (New England Biolabs) and 2.5 mM MgCl_2_. Cells were sonicated, then centrifuged at 15 000 × g for 30 min at 4°C. The supernatant was filtered and loaded onto a HisTrap FF 1 ml nickel column (GE Life Sciences). The column was washed first with 100% buffer A, then 3% buffer B (50 mM HEPES‐KOH, 300 mM KCl, 1 M imidazole, 10% glycerol, pH 7.2) to remove nonspecifically bound proteins. The His_6_‐tagged protein eluted on addition of 30% buffer B. Peak fractions were concentrated, then simultaneously incubated with His_6_‐ULP protease to cleave the His_6_‐SUMO tag and dialyzed overnight into buffer A. The solution was again loaded onto a HisTrap FF 1 ml nickel column and the flow through was collected, concentrated and dialyzed overnight into storage buffer (50 mM HEPES‐KOH, 300 mM KCl, 10% glycerol, pH 7.2), before being snap‐frozen in liquid nitrogen and stored at −80°C.

### Crystallization and structure determination

Initial conditions were identified at the MRC‐LMB crystallization facility (Stock *et al*., 2005). His_6_‐FzlA yielded cubic crystals in the following conditions: 50% PEG 200, 0.2 M NaCl, 0.1 M sodium potassium phosphate pH 6.2. Crystals diffracted to 2 Å and a native dataset was collected in house using a Rigaku X‐ray generator. Heavy metal derivatives were obtained by soaking the native crystals with ethyl mercury thiosalicylate (EMTS). The soaked crystals diffracted to 3 Å in house. FzlA crystals belonged to spacegroup I2_1_3 with one molecule in the asymmetric unit. Cell constants were *a* = *b* = *c* = 124.33 Å. Initial phases were determined with autoSHARP (Vonrhein *et al*., 2007). The model was built with MAIN (Turk, 2013) and refined with PHENIX (Adams *et al*., 2010). For more details please refer to Table 1. The structure was deposited in the Protein Data Bank (PDB) with code 5NR1.

### Bacterial two‐hybrid (BTH) analysis

Bacterial two‐hybrid testing was performed as described by Karimova et al (Karimova *et al*., 1998). Briefly, we constructed plasmids containing genes of interest fused to the T18 and T25 domains of the adenylate cyclase gene, which were then transformed into BTH101 *E. coli* cells. Transformants were grown overnight in liquid culture with 0.5 mM IPTG, then plated on agar containing 0.5 mM IPTG and 40 μg/ml X‐gal. In strains where the T18 and T25 fusions interacted, expression of β‐galactosidase resulted in blue colonies.

### Co‐immunoprecipitation

The co‐immunoprecipitation was performed as previously described (Bowman *et al*., 2008) without crosslinking and with a few other notable differences. Cell cultures were grown to exponential phase in 1 l of PYE medium, then pelleted. Pellets were washed and re‐suspended in co‐immunoprecipitation buffer [20 mM HEPES pH 7.5, 100 mM NaCl, 20% glycerol, Pierce Protease Inhibitor Tablet (1 tablet/l)]. The cell suspension was passed through a French press at 16 000 psi three times to achieve lysis. Membranes were solubilized by the addition of IGEPAL CA‐630 (1%), sodium deoxycholate (0.5%) and 2 mM EDTA. 3xFLAG‐FzlA was then immunoprecipitated using anti‐FLAG affinity agarose gel (Sigma), and bound proteins were eluted using excess FLAG peptide (Sigma). Samples were subjected to immunoblot analysis (see antibodies and immunoblotting section below).

### FzlA mutant gene library creation

A library of *fzlA* point mutants was created by first compiling a list of potential FtsZ‐interacting residues based on a number of criteria. Residues chosen were generally surface‐exposed, charged, conserved across α‐proteobacteria or a combination of these factors. Mutagenesis was performed using a QuikChange Lightning Multi Site‐Directed Mutagenesis Kit (Agilent Genomics). Primers were designed using Agilent's QuikChange Primer Design Program and contained 1–4 mutated residues each. The resulting mutations were nonconservative, containing either charge reversals (for charged residues) or dissimilar amino acids (eg, Ala for Trp). Mutations were made in pEG910 (pVCHYN‐2 *fzlA*), a plasmid encoding a vanillate‐inducible *mCherry‐fzlA* fusion, for use in *in vivo* screening.

### Light microscopy

Cells were imaged during log phase of growth on 1% agarose pads, as previously described (Meier *et al*., 2016). Microscopy was done using a Nikon Eclipse Ti inverted microscope with a Nikon Plan Fluor × 100 (numeric aperture 1.30) oil Ph3 objective and Photometrics CoolSNAP HQ^2^ cooled CCD (charge‐coupled device) camera. For fluorescence, an ET‐dsRED filter cube was used for mCherry and ET‐EYFP for Venus. For timelapse, isolated swarmer cells were placed on 1% agarose pads made with PYE after synchrony. Phase contrast images were taken every 5 min for 3 h.

### Image analysis

Images were processed in Adobe Photoshop. Automated cell length analysis was performed on unsynchronized cells using MicrobeJ (Ducret *et al*., 2016). Principle component analysis was performed on synchronized cells using Celltool (Pincus and Theriot, 2007). ImageJ was used to create binary masks from phase contrast images. These were used by Celltool to first create individual cell contours, which were then averaged to create a mean shape model of WT and mutant *fzlA* allelic exchange cells. The model created accounts for 97.3% of the variation in shape among cells in these populations. Maximum pole curvature of synchronized cells was quantified via Celltool, using a plugin created by the Pincus lab (Washington Univeristy‐St. Louis). Specifically, the maximum instantaneous curvature (the inverse of the radius of the oscillating disc) at the poles was determined and averaged across the two poles for each individual cell.

Timelapse images were analyzed using MicrobeJ. Initiation of constriction was automatically detected based on changes in positive curvature near midcell. Cells were tracked during constriction and were manually segmented on division. Cell width at midcell and cell length were recorded for each individual cell at each time point over the entire experiment. These data were used to calculate constriction time (number of frames from constriction initiation to division times five minutes per frame), constriction rate (starting cell width at initiation of constriction divided by constriction time), elongation rate before constriction (change in length before constriction divided by preconstriction time) and elongation rate during constriction (change in length from constriction initiation to division divided by constriction time).

### Antibodies and immunoblotting

For immunoblotting of whole cell lysates, log phase cells were pelleted, then resuspended and boiled in SDS‐PAGE loading buffer. For co‐immunoprecipitation, eluted samples were boiled in SDS‐PAGE loading buffer. Samples were resolved by SDS‐PAGE, and protein was subsequently transferred to nitrocellulose membranes. Membranes were first probed with α‐FzlA (Goley *et al*., 2010b) (1:8000), α‐HU (Bowman *et al*., 2010) (1:50 000; loading control), α‐FtsZ (1:20 000) or α‐RFP (1:2000) primary antibodies, then incubated with HRP‐conjugated α‐rabbit (1:20 000) secondary antibody, or probed with α‐FLAG (Sigma) (1:1000) primary antibody, then incubated with HRP‐conjugated α‐mouse (1:10 000) secondary antibody. Membranes were subsequently imaged with an Amersham Imager 600 RGB gel and membrane imager (GE).

### FtsZ activity assays

All biochemical assays were performed with 2 µM FtsZ and 4 µM FzlA in MESK polymerization buffer (50 mM MES [pH 6.5], 50 mM KCl) unless indicated otherwise. For high speed co‐sedimentation assays, unless otherwise indicated, FzlA ± FtsZ in MESK polymerization buffer containing 10 mM MgCl_2_, 2 mM GTP and 0.05% Triton X‐100 were incubated in triplicate for 15 min at room temperature, then were centrifuged at 280 000 × g for 15 min at 25°C. Low speed co‐sedimentation assays were similarly performed, but solutions instead contained 2.5 mM MgCl_2_ and were centrifuged at 16 000 × g for 15 min at room temperature. Pellet and supernatant from each sample were resolved by SDS‐PAGE and stained with Coomassie Brilliant Blue. Gels were imaged with a Gel Doc EZ Gel Imaging System (BioRad) and band intensity was quantified using Image Lab (BioRad). Band intensities were used to calculate the percentage of FzlA present in the pellet.

RALS of FzlA ± FtsZ in MESK polymerization buffer with 2.5 mM MgCl_2_ and 2 mM GTP was measured using a Fluoromax‐3 spectrofluorometer (Jobin Yvon Inc.) with 350 nm excitation and emission wavelengths and 2 nm slits. TEM was performed on FzlA ± FtsZ in MESK polymerization buffer with 2.5 mM MgCl_2_ and 2 mM GTP using 0.75% uranyl formate staining as previously described (Sundararajan *et al*., 2015). TEM Samples were imaged using a Philips/FEI BioTwin CM120 TEM equipped with an AMT XR80 8 megapixel CCD camera (AMT Imaging, USA). GTPase activity of FtsZ (1.75 μm) ± WT or mutant FzlA protein (3.5 μm) was measured in triplicate using a colorimetric SensoLyte MG Phosphate Assay Kit (AnaSpec), following the company's protocol.

## Conflict of Interest

We declare no competing interest.

## Author Contributions

P.J.L., P.S., C.R.M., J.L. and E.D.G. planned the experiments. P.J.L., C.R.M. and P.S. performed the experiments. P.J.L., P.S., C.R.M. and E.D.G. wrote the manuscript. P.J.L., P.S., C.R.M., J.L. and E.D.G. edited the manuscript.

## Supporting information

Supporting Figures and Table

Supporting Table2

Supporting Table3

Supporting Video1

Supporting Video2

Supporting Video3

Supporting Video4
