# Supplementary material for: FzlA, an essential regulator of FtsZ filament curvature, controls constriction rate during Caulobacter division
Source: Mol Microbiol. 2017 Dec 1;107(2):180–97. doi: 10.1111/mmi.13876 (PMC5760450; doi:10.1111/mmi.13876)
Supplement: Supplementary file 1 — Supporting Figures and Table [file MMI-107-180-s001.pdf]

FzlA, an essential regulator of FtsZ filament curvature, controls constriction rate during *Caulobacter* division

Lariviere *et al.*

Supporting information

Supplemental Discussion

Attempts to co-crystallize FtsZ and FzlA were unsuccessful

We attempted to co-crystallize FtsZ and FzlA at the MRC-LMB crystallization facility. His_6-FzlA was mixed with either full length FtsZ or FtsZΔCTL. We expected that full length FtsZ might not crystallize readily due to flexibility of the linker domain and we had found that FzlA interacts with FtsZΔCTL. Protein mixtures were made containing either a 1:1 ratio of FtsZ:FzlA or an excess of either FtsZ or FzlA, then incubated at room temperature or 4°C to allow for crystal growth. The only crystals that could be found were FzlA alone. For these experiments, His_6-FzlA and FtsZ/FtsZΔCTL were purified individually, as described in the methods section.

The P_{van} promoter is leaky

We were curious if any mutant FzlA screening strains could grow in the absence of either inducer. We found when we grew cells in the absence of xylose or vanillate that numerous mutant strains had cells that became elongated and had reduced viability (*$fzlA^{WB1}$, $fzlA^{UE1}$-$fzlA^{UE2}$, $fzlA^{NB1}$-$fzlA^{NB2}$, $fzlA^{NH1}$, $fzlA^{W9}$, $fzlA^{ST1}$-$fzlA^{ST7}$, $fzlA^{SOL1}$-$fzlA^{SOL6}$*), as expected (**Figure S3**). Surprisingly, however, WT cells and many of the mutant strain cells exhibited WT cell length and were viable (*$fzlA^{UN1}$, $fzlA^{NH2}$-$fzlA^{NH3}$, $fzlA^{W1}$-$fzlA^{W8}$,* and *$fzlA^{W10}$*) (**Figure S3**). α-FzlA immunoblotting of the mutants that were subsequently characterized biochemically revealed that in the absence of inducers, low levels of the mCherry-FzlA variants and WT FzlA were present (**Figure S9**). This suggests that the P_{van}, and to a lesser extent P_{xyl}, promoters were leaky (**Figure S4B**). The low expression levels of the functional or partially functional mutant FzlA variants were apparently sufficient to enable growth.

Untagged FzlA forms helices with FtsZ at pH 6.5

His_6-FzlA was previously shown to bind, form helices with, and lower the GTPase rate of FtsZ using HEK polymerization buffer (50 mM HEPES-KOH [pH7.2], 50 mM KCl, 0.1 mM EDTA) with 2.5 mM $MgCl_2$ and 2 mM GTP. For our study, we sought to characterize the activity of untagged FzlA mutant proteins, in order to control against any potential His_6 artifacts. We purified His_6-SUMO-WT FzlA, then cleaved the affinity tag. Interestingly, when we conducted the FtsZ interaction assays with untagged FzlA using HEK polymerization buffer and the same conditions as before, although FzlA still bound FtsZ efficiently, we rarely observed FtsZ helix formation or an effect on GTPase rate. After optimizing numerous variables, we found that untagged FzlA promotes formation of FtsZ helices efficiently at pH 6.5, but not pH 7.2 (data not shown). This may reflect the difference in the predicted pI of the two forms of FzlA: His_6-FzlA is predicted to have a pI of 7.4 and untagged FzlA is predicted to have a pI of 6.8. Untagged FzlA would therefore be above its pI at pH 7.2 whereas the His_6-tagged version would be below its pI. Lowering the pH to 6.5 would be predicted to place both proteins below their pI and

render each a net positive charge. Since FtsZ still efficiently polymerizes and hydrolyzes GTP at this lower pH, we carried out all FzlA-FtsZ interaction assays using these conditions.

Although the pH and salt concentration of the *E. coli* cytoplasm has been reported to be close to neutral and 250 mM potassium, respectively, there is no information on the conditions of the *C. crescentus* cytoplasm. Given the distinct environmental niches occupied by *E. coli* and *C. crescentus* it may not be fair to assume equivalent pH and salt concentrations in *C. crescentus*. Therefore, it is difficult to know what the appropriate "physiological" conditions might be to inform conditions for our *in vitro* assays. In order to maximize our ability to distinguish possible differences between our mutant FzlA proteins and WT, we used pH 6.5, 50 mM KCl buffer for all of our *in vitro* assays. We acknowledge that it is impossible to fully recapitulate the chemical and physical conditions of the cell – even when these are known - in a test tube, so all activities observed *in vitro* must be viewed with the caveat that they may not occur in the context of the cell.

Table S1. Additional FzlA mutant phenotypes and activity

Mutant Class	Name	Residue Mutations	P_{van} *mChy-mutant fzlA*, P_{xyl} *fzlA* Doubling Time (min)	Max OD_{600}	FzlA Localization
Poor **St**ability	$FzlA^{ST1}$	E25K	-	-	No signal
	$FzlA^{ST2}$	R94D E96K R98D	-	-	Diffuse
	$FzlA^{ST3}$	E151K K188D D189R	-	-	No signal
	$FzlA^{ST4}$	W159A W196A K199D	-	-	Diffuse
	$FzlA^{ST5}$	G183W V185A	122.9 ± 2.9	1.85 ± 0.0327	Diffuse
	$FzlA^{ST6}$	W187A K194E	-	-	Diffuse
	$FzlA^{ST7}$	K201D S202A R203D P204A	-	-	Diffuse
Poor **Sol**ubility	$FzlA^{SOL1}$	R20D	-	-	Diffuse
	$FzlA^{SOL2}$	R99D W103A D105K	-	-	Diffuse
	$FzlA^{SOL3}$	D109R R127D	133.0 ± 0.7	2.24 ± 0.0355	Diffuse
	$FzlA^{SOL4}$	E111K H146D	150.9 ± 4.2	0.85 ± 0.0425	Diffuse
	$FzlA^{SOL5}$	W187A	-	-	Diffuse
	$FzlA^{SOL6}$	R207D L210A	144.6 ± 0.6	1.63 ± 0.0225	Midcell
**W**T-like	$FzlA^{W1}$	P14A	-	-	Midcell
	$FzlA^{W2}$	R27D E81K E83K	-	-	Midcell
	$FzlA^{W3}$	K60D H61D R63D	-	-	Midcell
	$FzlA^{W4}$	R89D D90R	-	-	Midcell
	$FzlA^{W5}$	K120D D184R	-	-	Midcell
	$FzlA^{W6}$	D132R	-	-	Midcell
	$FzlA^{W7}$	L153A R157D	-	-	Midcell
	$FzlA^{W8}$	D158R R163D R164D	-	-	Midcell
	$FzlA^{W9}$	D212R R213D	-	-	Midcell
	$FzlA^{W10}$	P219A H222D	-	-	Midcell
	$FzlA^{W11}$	F228A	-	-	Midcell
**U**nknown **N**on-essential activity	$FzlA^{UN2}$	Y223A	-	-	Midcell

Values shown as mean ± SEM. For co-pelleting data, values are reported as fold change of % FzlA in pellet alone vs. with FtsZ.

Table S2. Plasmid List (see excel file)

Table S3. Strain List (see excel file)

Supplemental Figures

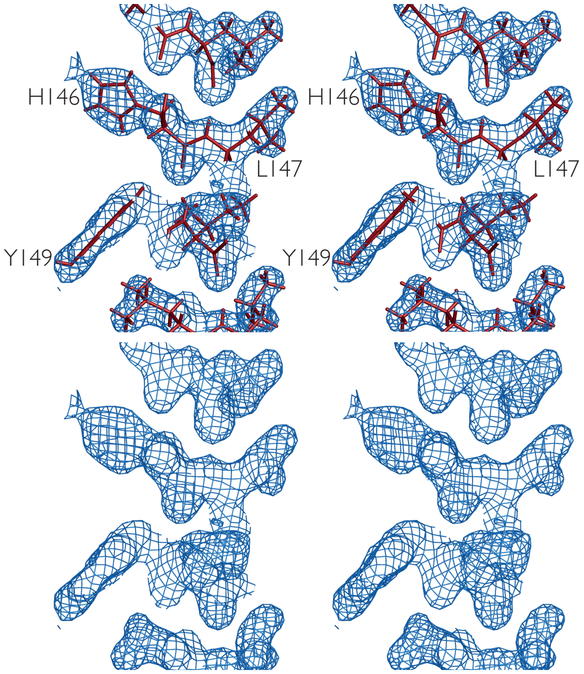

Fig. S1. Close-up of a stereo image of the electron density map of FzlA
Shown are wall-eyed stereo images of the 2Fo-Fc map contoured at the sigma level 1.0. Views of the map with and without fitted atomic model are presented next to each other displaying H146, L147 and Y149.

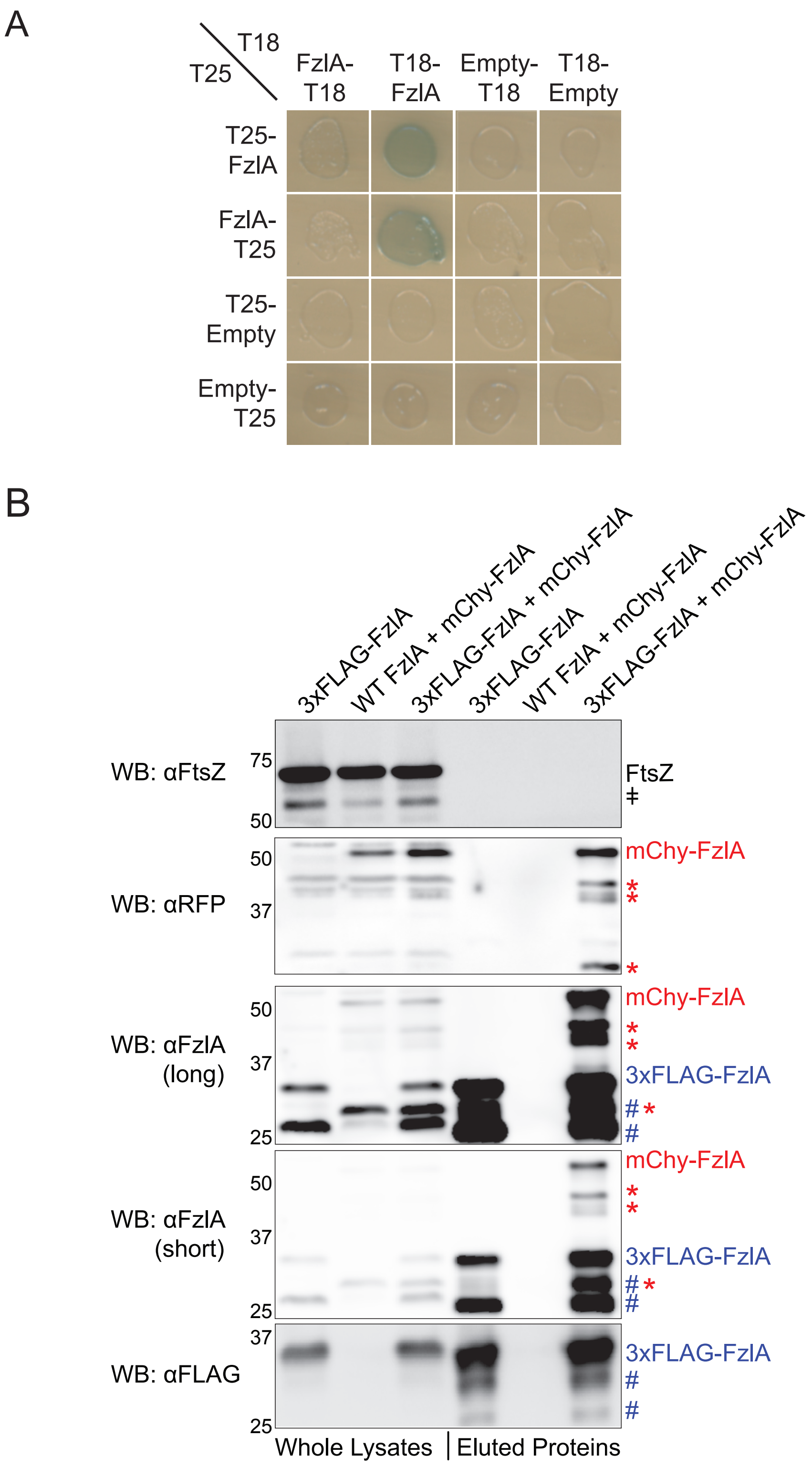

Fig. S2. FzlA self-interacts *in vivo*

A. Cultures of *E. coli* BTH101 strains producing the indicated FzlA fusions or carrying empty vector controls spotted on plates containing IPTG and X-Gal. T18-FzlA interacts with both N- and C-terminal T25 FzlA fusions. B. mCherry-FzlA co-immunoprecipitates with 3xFLAG-FzlA

from *C. crescentus* cell lysates. Western blot analysis of whole cell lysates of strains producing the indicated FzlA variants (left) and eluates from samples of the same strains obtained by immunoprecipitation with α-FLAG resin and elution with FLAG peptide (right). Cultures were grown to log phase and induced for 1hr with vanillate before preparation of lysates and immunoprecipitation. Antibodies used for western blotting (WB) are indicated to the left of the blots. Long=longer exposure. Short = shorter exposure. ‡ = FtsZ degradation product. Red = mCherry-FzlA derived species. * = mCherry-FzlA degradation products. Blue = 3xFLAG-FzlA derived species. # = 3xFLAG-FzlA degradation products. mChy = mCherry. Strain key: 3xFLAG-FzlA (EG2217), WT FzlA + mChy-FzlA (EG2451), 3xFLAG-FzlA+ mChy-FzlA (EG2452).

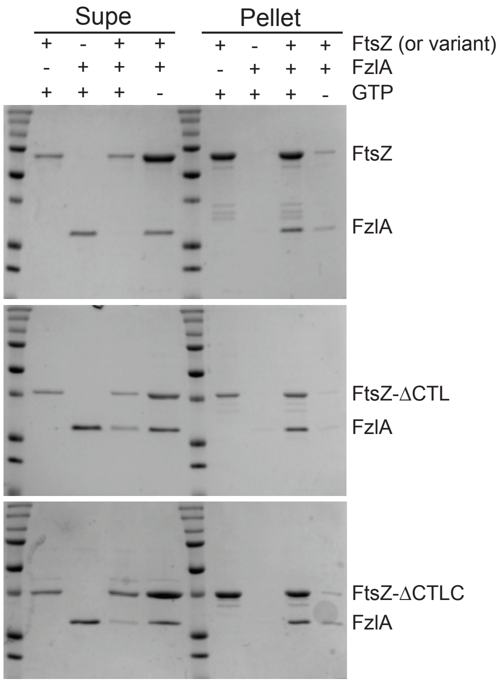

Fig. S3. FzlA Interacts with the GTPase domain of FtsZ

Coomassie-stained SDS-PAGE gels of supernatant and pellet fractions from high-speed co-sedimentation assays of 3 μM His$_6$-FzlA + 3 μM FtsZ (top), 3 μM FtsZΔCTL (middle), and 3 μM FtsZΔCTLC (bottom). Both His$_6$-FzlA and each FtsZ variant are preferentially recruited to the pellet in the presence of GTP. Reactions were performed in HEK50 polymerization buffer (50 mM HEPES-KOH [pH 7.2], 50 mM KCl, 0.1 mM EDTA) with 2 mM GTP, 2.5 mM $MgCl_2$, and 0.05% Triton X-100.

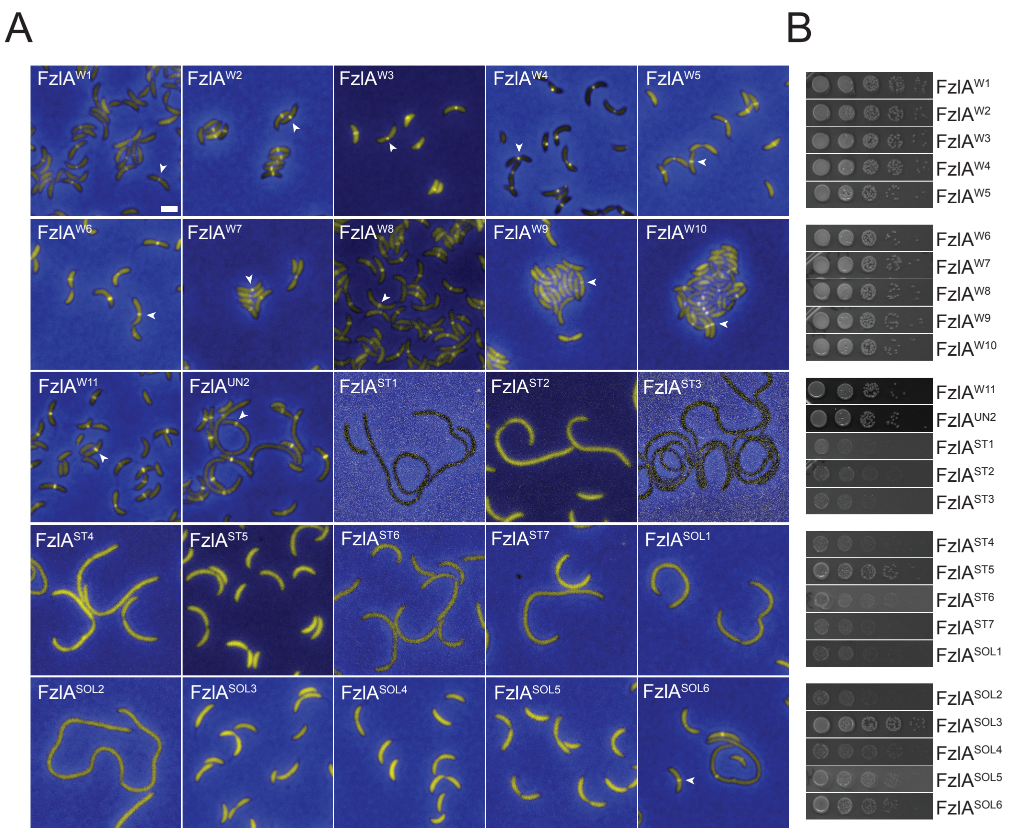

Fig. S4. Many *fzlA* mutant strains displayed wild-type phenotypes or were unstable or insoluble.

A. Merged fluorescence microscopy images depicting mCherry-FzlA mutant protein (yellow) localization in cells in Δ*fzlA* P_{xyl}-*fzlA* P_{van}-*mCherry-fzlA* mutant backgrounds (labels indicate mutant alleles fused to mCherry, with expression driven by P_{van}). Cells were depleted of WT FzlA and grown with vanillate for 24 hours prior to imaging. White arrowheads mark localized FzlA bands. Scale bar = 2 µm. B. Spot dilutions of Δ*fzlA* P_{xyl}-*fzlA*, P_{van}-*mCherry-fzlA* mutant strains, plated on PYE-agar with vanillate. Strain key: FzlA^{W1} (EG1426), FzlA^{W2} (EG1428), FzlA^{W3} (EG1431), FzlA^{W4} (EG1432), FzlA^{W5} (EG1439), FzlA^{W6} (EG1440), FzlA^{W7} (EG1443), FzlA^{W8} (EG1444), FzlA^{W9} (EG1455), FzlA^{W10} (EG1456), FzlA^{W11} (EG2304), FzlA^{UN2} (EG2302), FzlA^{ST1} (EG1429), FzlA^{ST2} (EG1433), FzlA^{ST3} (EG1454), FzlA^{ST4} (EG1450), FzlA^{ST5} (EG1451), FzlA^{ST6} (EG1314), FzlA^{ST7} (EG1452), FzlA^{SOL1} (EG1427), FzlA^{SOL2} (EG1434), FzlA^{SOL3} (EG1436), FzlA^{SOL4} (EG1437), FzlA^{SOL5} (EG1315), FzlA^{SOL6} (EG1453).

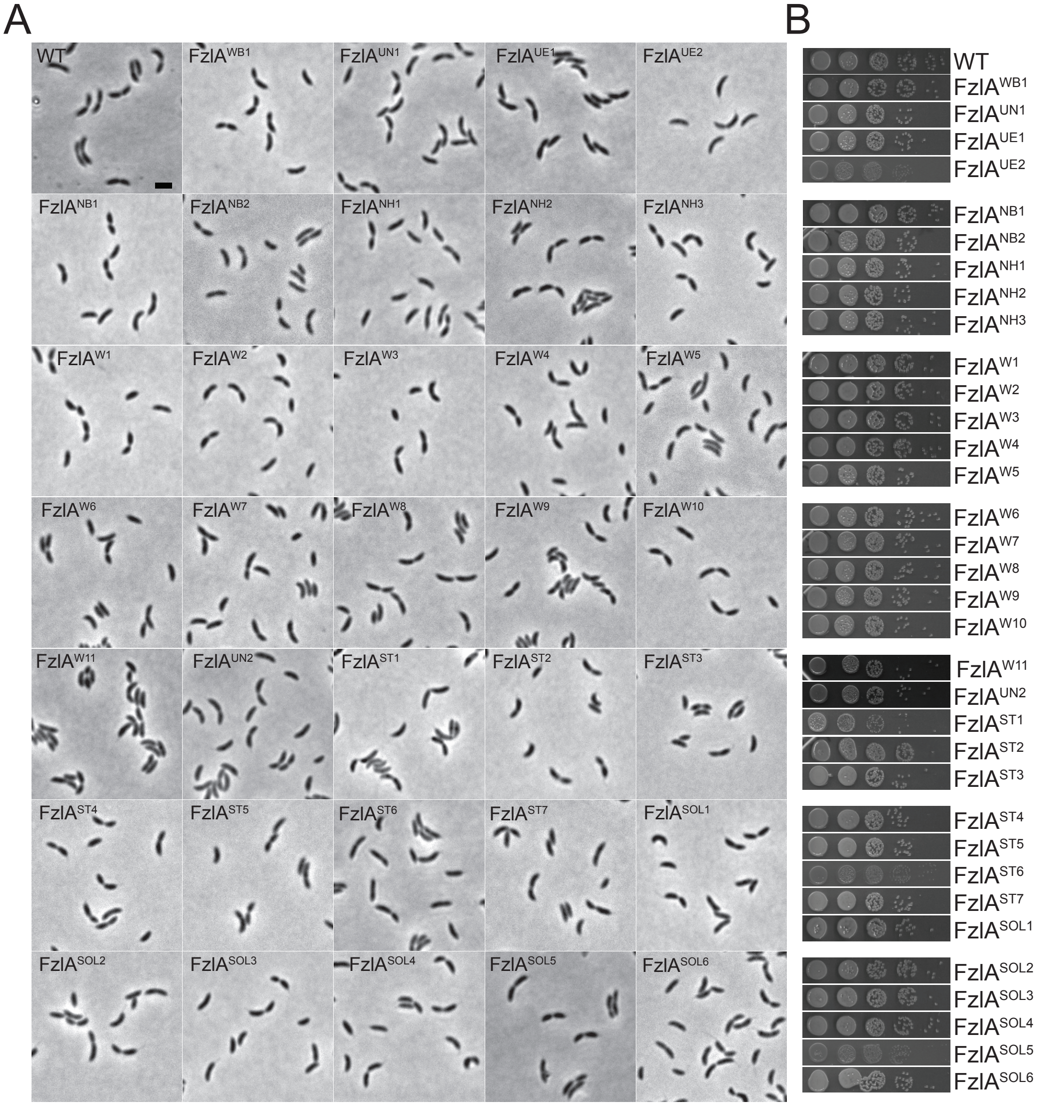

Fig. S5. *fzlA* mutant strains grown with xylose to induce WT FzlA grow normally.
A. Phase contrast images depicting mutant cells with Δ*fzlA* P$_{xyl}$-*fzlA* P$_{van}$-*mCherry-fzlA* mutant backgrounds. Cells were grown with xylose. Scale bar = 2 µm. B. Spot dilutions of Δ*fzlA* P$_{xyl}$-*fzlA* P$_{van}$-*mCherry-fzlA* mutant strains, plated on PYE-agar with xylose. Strain key: WT (NA1000), FzlA^{WB1} (EG1435), FzlA^{UN1} (EG1312), FzlA^{UE1} (EG1313), FzlA^{UE2} (EG1621), FzlA^{NB1} (EG1430), FzlA^{NB2} (EG1438), FzlA^{NH1} (EG1311), FzlA^{NH2} (EG1441), FzlA^{NH3} (EG1442), FzlA^{W1} (EG1426), FzlA^{W2} (EG1428), FzlA^{W3} (EG1431), FzlA^{W4} (EG1432), FzlA^{W5} (EG1439), FzlA^{W6} (EG1440), FzlA^{W7} (EG1443), FzlA^{W8} (EG1444), FzlA^{W9} (EG1455), FzlA^{W10} (EG1456), FzlA^{W11} (EG2304), FzlA^{UN2} (EG2302), FzlA^{ST1} (EG1429), FzlA^{ST2} (EG1433), FzlA^{ST3} (EG1454), FzlA^{ST4} (EG1450), FzlA^{ST5} (EG1451), FzlA^{ST6} (EG1314), FzlA^{ST7} (EG1452), FzlA^{SOL1} (EG1427), FzlA^{SOL2} (EG1434), FzlA^{SOL3} (EG1436), FzlA^{SOL4} (EG1437), FzlA^{SOL5} (EG1315), FzlA^{SOL6} (EG1453).

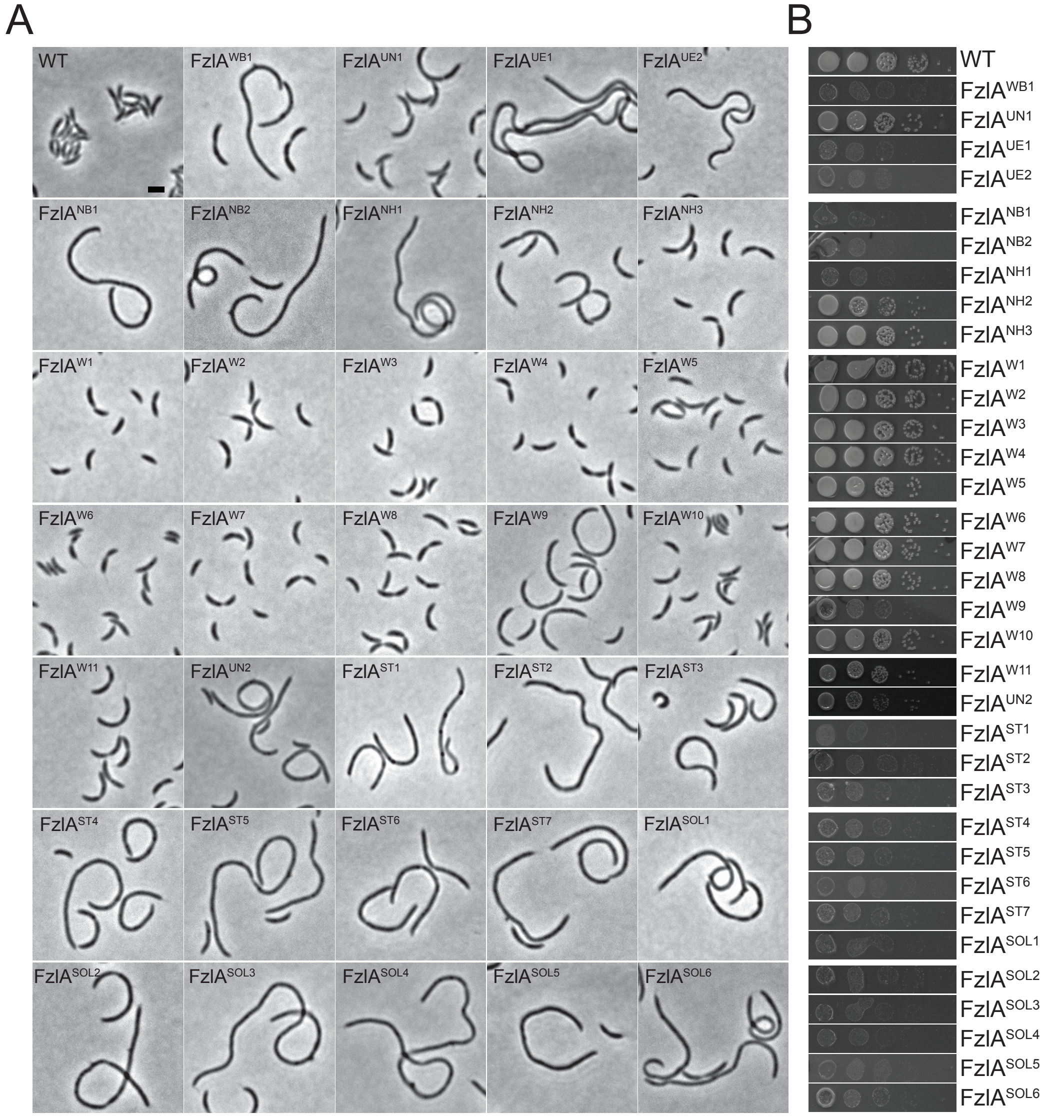

Fig. S6. *fzlA* mutant strains grown without inducers display a range of growth phenotypes that correlate with their activities in the presence of vanillate.
A. Phase contrast images depicting mutant cells with Δ*fzlA* P$_{xyl}$-*fzlA* P$_{van}$-*mCherry-fzlA* mutant backgrounds. Cells were grown without inducers and with glucose to repress the P$_{xyl}$ promoter for 24 hours prior to imaging. (FzlA^{ST1} cells displayed severe growth defects and did not reach log phase in 24 hours. These cells were imaged after 48 hours.) Scale bar = 2 µm. Note that for WT cells and strains with mutant *fzlA* genes that support division, leaky expression from the P$_{van}$ promoter is sufficient to support growth. B. Spot dilutions of Δ*fzlA* P$_{xyl}$-*fzlA* P$_{van}$-*mCherry-fzlA* mutant strains, plated on PYE-ager with glucose and without inducers. Strain key: WT (NA1000), FzlA^{WB1} (EG1435), FzlA^{UN1} (EG1312), FzlA^{UE1} (EG1313), FzlA^{UE2} (EG1621), FzlA^{NB1} (EG1430), FzlA^{NB2} (EG1438), FzlA^{NH1} (EG1311), FzlA^{NH2} (EG1441), FzlA^{NH3} (EG1442), FzlA^{W1} (EG1426), FzlA^{W2} (EG1428), FzlA^{W3} (EG1431), FzlA^{W4} (EG1432), FzlA^{W5} (EG1439), FzlA^{W6} (EG1440), FzlA^{W7} (EG1443), FzlA^{W8} (EG1444), FzlA^{W9} (EG1455), FzlA^{W10} (EG1456), FzlA^{W11} (EG2304), FzlA^{UN2} (EG2302), FzlA^{ST1} (EG1429), FzlA^{ST2} (EG1433), FzlA^{ST3} (EG1454), FzlA^{ST4} (EG1450), FzlA^{ST5} (EG1451), FzlA^{ST6} (EG1314), FzlA^{ST7}

(EG1452), FzlA^{SOL1} (EG1427), FzlA^{SOL2} (EG1434), FzlA^{SOL3} (EG1436), FzlA^{SOL4} (EG1437), FzlA^{SOL5} (EG1315), FzlA^{SOL6} (EG1453).

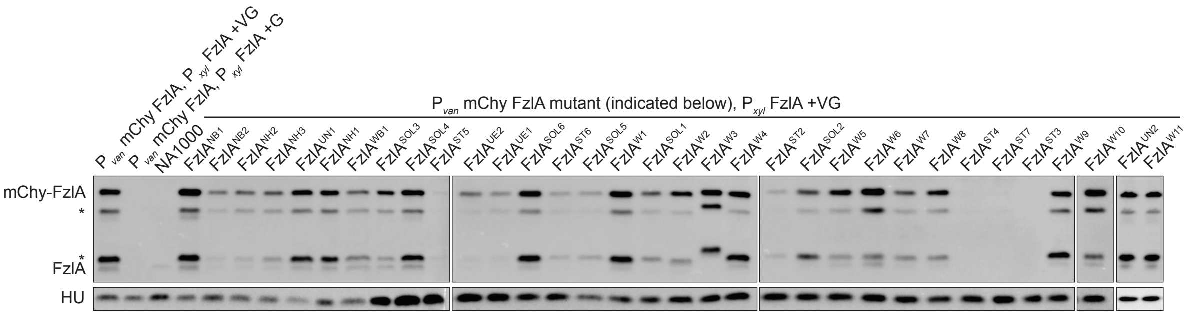

Fig. S7. Protein levels of mCherry-FzlA under induction conditions.

α-FzlA (top) and α-HU (bottom, loading control) immunoblot of Δ*fzlA* P_{xyl}-*fzlA* P_{van}-*mCherry-fzlA* mutant strain lysates depleted of WT FzlA and grown for 24 hours with vanillate and glucose. *=degradation product. V = vanillate, G = glucose. The strain containing *mCherry-fzlA*^{ST1} was severely sick and did not reach log phase in 24 hours. It was therefore not included in immunoblot analysis. Strain key: P_{van} mChy FzlA, P_{xyl} FzlA (EG1310), FzlA^{NB1} (EG1430), FzlA^{NB2} (EG1438), FzlA^{NH2} (EG1441), FzlA^{NH3} (EG1442), FzlA^{UN1} (EG1312), FzlA^{NH1} (EG1311), FzlA^{WB1} (EG1435), FzlA^{SOL3} (EG1436), FzlA^{SOL4} (EG1437), FzlA^{ST5} (EG1451), FzlA^{UE2} (EG1621), FzlA^{UE1} (EG1313), FzlA^{SOL6} (EG1453), FzlA^{ST6} (EG1314), FzlA^{SOL5} (EG1315), FzlA^{W1} (EG1426), FzlA^{SOL1} (EG1427), FzlA^{W2} (EG1428), FzlA^{W3} (EG1431), FzlA^{W4} (EG1432), FzlA^{ST2} (EG1433), FzlA^{SOL2} (EG1434), FzlA^{W5} (EG1439), FzlA^{W6} (EG1440), FzlA^{W7} (EG1443), FzlA^{W8} (EG1444), FzlA^{ST4} (EG1450), FzlA^{ST7} (EG1452), FzlA^{ST3} (EG1454), FzlA^{W9} (EG1455), FzlA^{W10} (EG1456), FzlA^{UN2} (EG2302), FzlA^{W11} (EG2304).

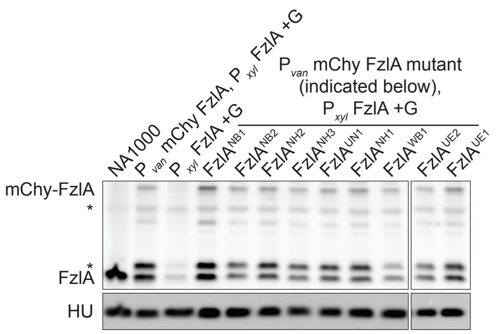

Fig. S8. Protein levels of mCherry-FzlA under depletion conditions.
α-FzlA (top) and α-HU (bottom, loading control) immunoblot of Δ*fzlA* P$_{xyl}$-*fzlA* P$_{van}$-*mCherry-fzlA* mutant strain lysates depleted of WT FzlA and grown for 24 hours with glucose. *=degradation product. G = glucose. Only strains that were biochemically characterized are shown. Strain key: NA1000 (WT), P$_{van}$ mChy FzlA, P$_{xyl}$ FzlA (EG1310), P$_{xyl}$ FzlA (EG312), FzlA^{NB1} (EG1430), FzlA^{NB2} (EG1438), FzlA^{NH2} (EG1441), FzlA^{NH3} (EG1442), FzlA^{UN1} (EG1312), FzlA^{NH1} (EG1311), FzlA^{WB1} (EG1435),), FzlA^{UE2} (EG1621), FzlA^{UE1} (EG1313).

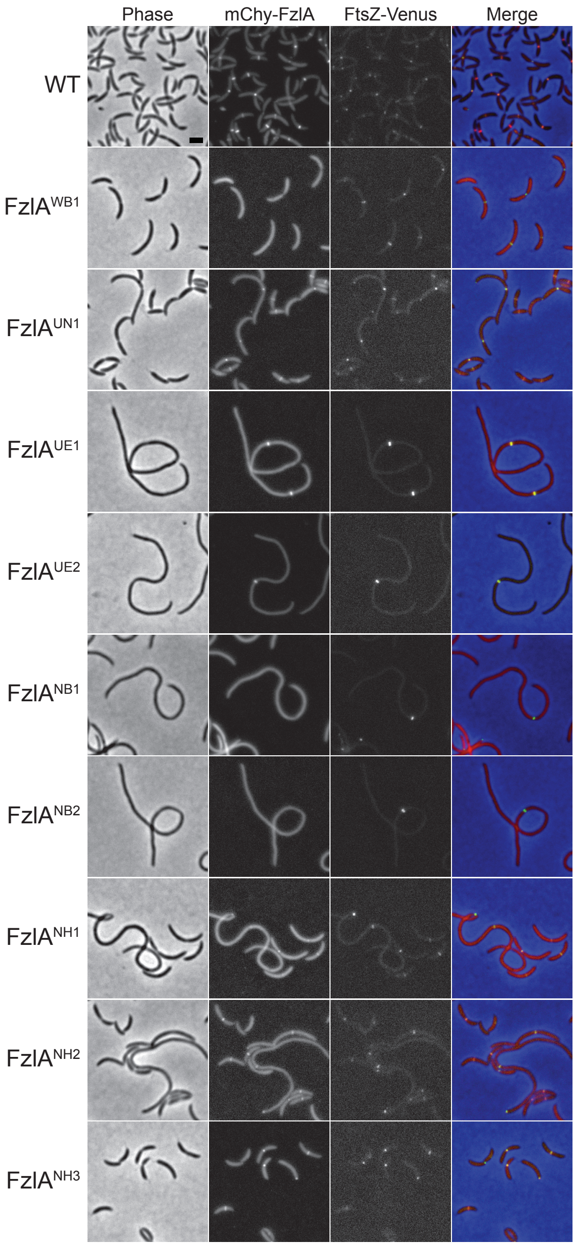

Fig. S9. FtsZ forms rings in cells expressing *fzlA* mutant alleles
Merged fluorescence microscopy images depicting mCherry-FzlA mutant protein (red) and FtsZ-Venus (green) localization in cells with Δ*fzlA* P_{xyl}-*fzlA* P_{van}-*mCherry-fzlA* mutant backgrounds that carry a low copy plasmid containing P_{lac}-FtsZ-Venus. Cells were depleted of WT FzlA and grown with vanillate for 24 hours prior to imaging. IPTG was not added as we found that leaky expression was sufficient for low levels of FtsZ-Venus expression. Yellow indicates co-localization of mCherry-FzlA and FtsZ-Venus. Scale bar = 2 μm. Strain key: WT (EG1760), FzlA^{WB1} (EG1762), FzlA^{UN1} (EG1628), FzlA^{UE1} (EG1767), FzlA^{UE2} (EG1766), FzlA^{NB1} (EG1761), FzlA^{NB2} (EG1765), FzlA^{NH1} (EG1770), FzlA^{NH2} (EG1629), FzlA^{NH3} (EG1768).

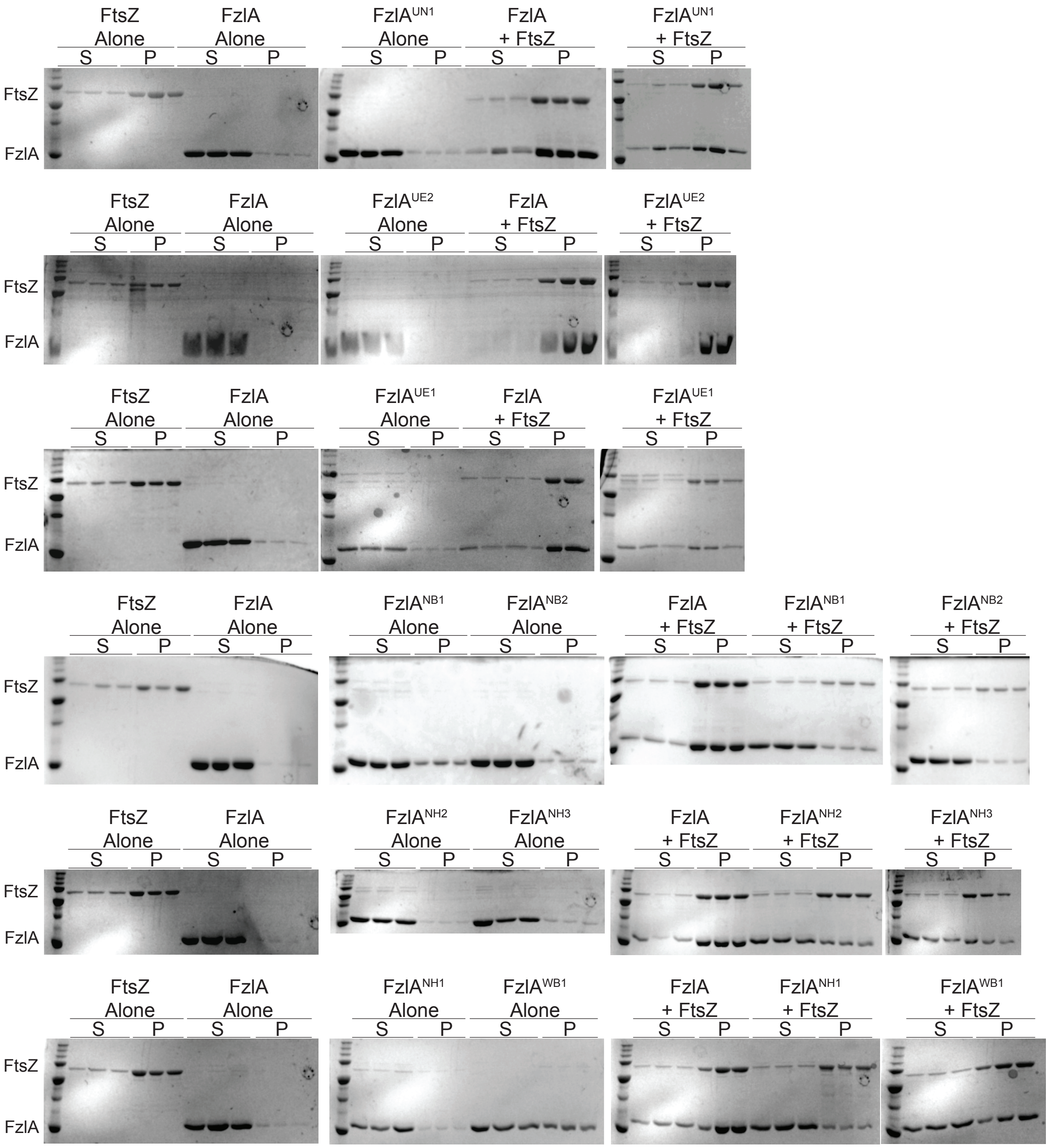

Fig. S10. High speed co-pelleting of FzlA mutant proteins and FtsZ

Coomassie-blue stained SDS-PAGE gels of FtsZ (2 μM) and FzlA mutant proteins (4 μM) that have been co-sedimented at high speed under polymerizing conditions, used for quantification (main text). S=supernatant, P=pellet. Experiments performed in triplicate, as shown. Each row shows samples run on the same day. Control experiments with FtsZ and WT FzlA were run each time for comparison to different FzlA mutants and to control for day-to-day variations.

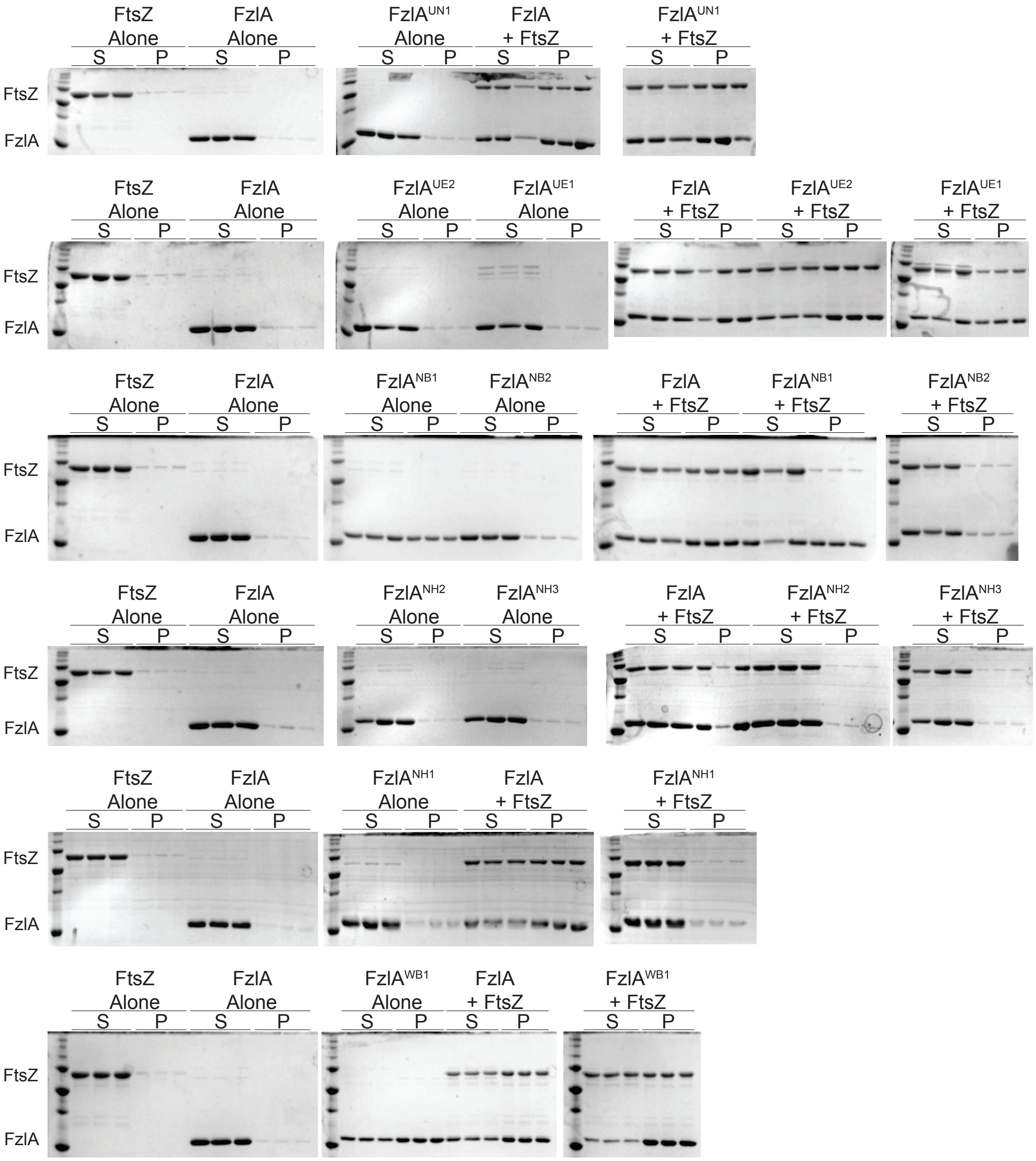

Fig. S11. Low speed co-pelleting of FzlA mutant proteins and FtsZ

Coomassie-blue stained SDS-PAGE gels of FtsZ (2 μM) and FzlA mutant proteins (4 μM) that have been co-sedimented at low speed under polymerizing conditions, used for quantification (main text). S=supernatant, P=pellet. Experiments performed in triplicate, as shown. Each row shows samples run on the same day. Control experiments with FtsZ and WT FzlA were run each time for comparison to different FzlA mutants and to control for day-to-day variations.

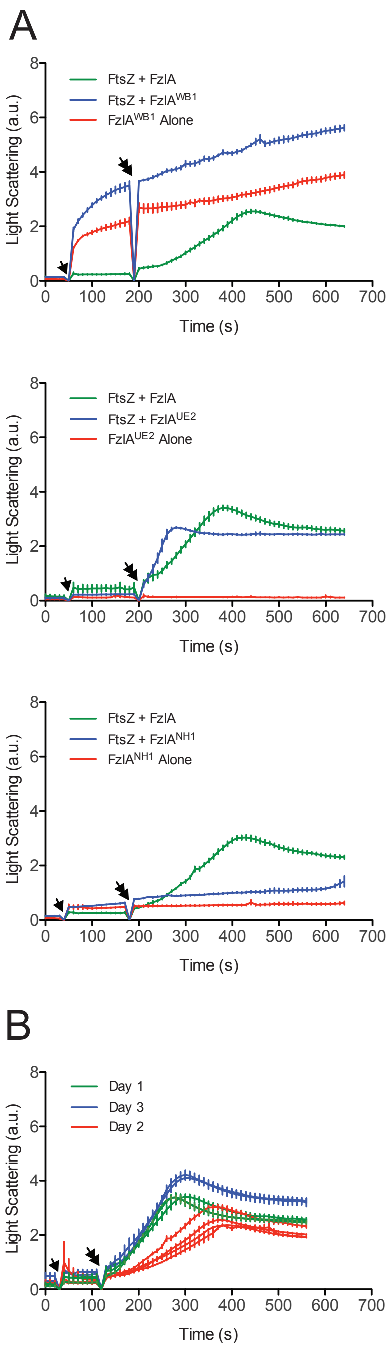

Fig. S12. Some FzlA mutant proteins are able to form higher order structures in the presence of FtsZ.

A. Plots of RALS over time for 4 µM FzlA WT or mutant protein ± 2 µM FtsZ. Mean ± SEM is shown. Cuvettes containing FtsZ received injections of FzlA mutant protein (single arrow), then 2 mM GTP (double arrow) to induce polymerization. Experiments were performed in triplicate (except for FzlA^{UE1} alone, which was done in duplicate). Some mutant proteins (WB1, NB1, NB2) displayed self-association on their own, as evidenced by an increase in signal without FtsZ (red lines). B. Plot of RALS over time for the FtsZ + FzlA control reaction for each experiment shown in panel A. Experiments were performed on three separate days, with the relevant mutant

proteins noted: Day 1 (FzlA^{UE1}, FzlA^{UE2}, FzlA^{UN1}), Day 2 (FzlA^{WB1}, FzlA^{Nh1}, FzlA^{NH2}, FzlA^{NH3}), and Day 3 (FzlA^{NB1}, FzlA^{NB2}). FtsZ + FzlA displayed mild variability in terms of rate of light scattering and maximum intensity, but the characteristic shape of the curve remained relatively constant.

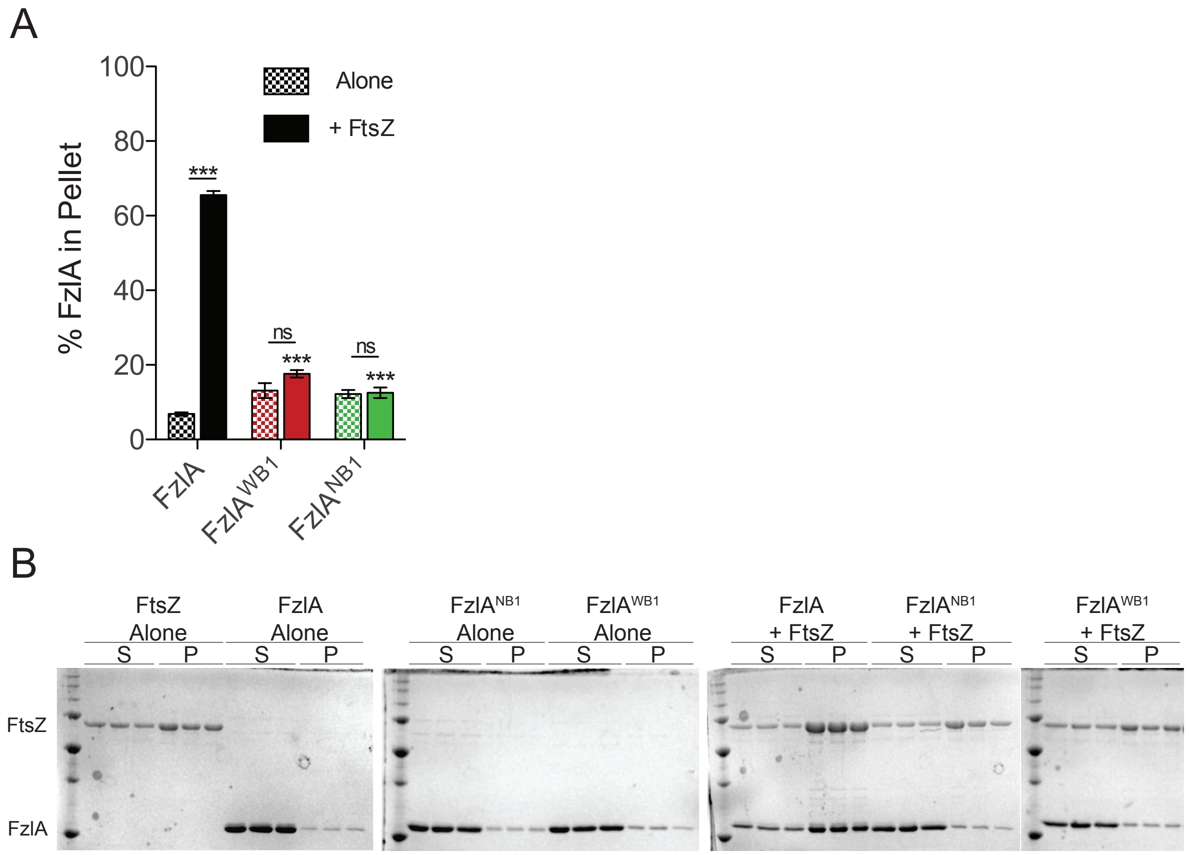

Fig. S13. High speed co-pelleting of FzlA^{WB1} or FzlA^{NB1} and FtsZ
A, B. Quantification of percent FzlA found in pellet (A) and Coomassie-blue stained SDS-PAGE gels (B) of FtsZ (4 µM) and FzlA mutant protein (4 µM) that have been co-sedimented at high speed in HEK50 polymerization buffer (50 mM HEPES, 50 mM KCl, pH 7.2; 2 mM GTP; 10 mM $MgCl_2$). For (A), a one way ANOVA with Dunnett's multiple comparison test was performed to assess differences between each FzlA mutant protein + FtsZ and WT FzlA + FtsZ (lower asterisks). Unpaired t-tests were performed to analyze the difference between a given FzlA mutant protein alone vs. with FtsZ. For all statistical tests, $^{ns}P>0.05$, $^{***}P\leq0.001$ and n=3 for each sample. For (B), S=supernatant, P=pellet.

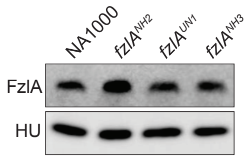

Fig. S14. Protein levels of FzlA in *fzlA* allelic exchange strains.
α-FzlA (top) and α-HU (bottom) immunoblots of *fzlA* allelic exchange strain lysates. Strain key: NA1000 (WT), *fzlA*^{NH2} (EG1600), *fzlA*^{UN1} (EG1671), *fzlA*^{NH3} (EG1909).

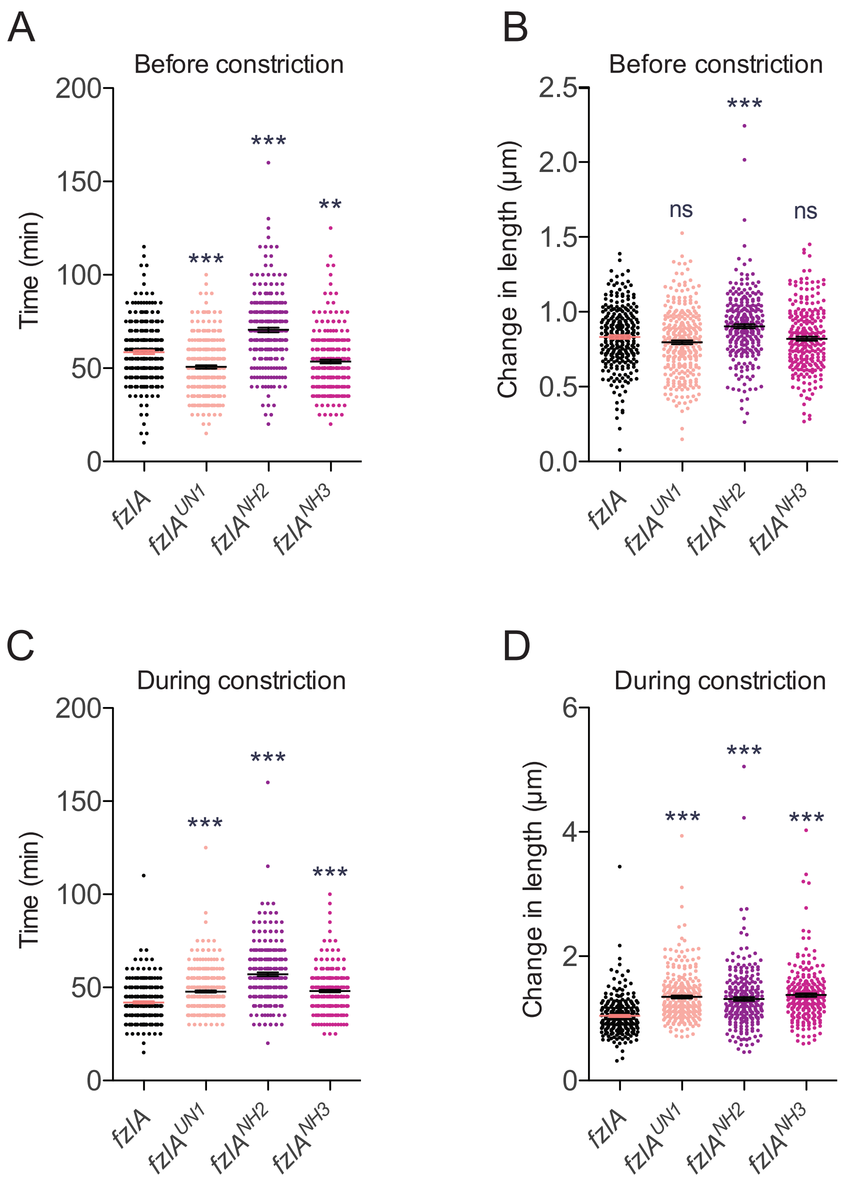

Fig. S15. Pre-constriction time and change in cell length for *fzlA* allelic exchange strains. A-D. Plots of pre-constriction time (A), change in cell length before constriction (B), constriction time (C) and change in cell length during constriction (D) for a population of synchronized cells from each *fzlA* strain, calculated from single cell microscopy data. Pre-constriction time was calculated by determining the number of frames from initial image acquisition until cells began to divide, then converting to minutes. Constriction time was calculated by counting the number of frames from constriction initiation until cell separation, then converting into minutes. Mean ± SEM is shown. A one way ANOVA with Tukey's multiple comparison test was performed to analyze differences compared to WT: $^{ns}P>0.05$, $^{**}P\leq0.01$, $^{***}P\leq0.001$. From left to right, n= 271, 295, 260, 249 (A, B, C) and 266, 293, 258, 246 (D). Strain key: *fzlA* (NA1000), *fzlA*^{UN1} (EG1908), *fzlA*^{NH2} (EG1600), *fzlA*^{NH3} (EG1909).

Videos 1-4. Timelapse movies of constricting *C. crescentus* cells.
Synchronized WT (1), *fzlA*^{UN1} (2), *fzlA*^{NH2} (3), or *fzlA*^{NH3} (4) cells grown on PYE and imaged every 5 minutes. Videos are shown at 8 fps (2400x speed).

Supplemental References

Aakre, C.D., Phung, T.N., Huang, D., and Laub, M.T. (2013) A Bacterial Toxin Inhibits DNA Replication Elongation Through a Direct Interaction with the β Sliding Clamp. *Mol Cell* **52**: 617–628.

Abel, S., Bucher, T., Nicollier, M., Hug, I., Kaever, V., Abel Zur Wiesch, P., and Jenal, U. (2013) Bi-modal distribution of the second messenger c-di-GMP controls cell fate and asymmetry during the caulobacter cell cycle. *PLoS Genet* **9**: e1003744.

Bendezú, F.O., Hale, C.A., Bernhardt, T.G., and Boer, P.A.J. de (2009) RodZ (YfgA) is required for proper assembly of the MreB actin cytoskeleton and cell shape in E. coli. *EMBO J* **28**: 193–204.

Evinger, M., and Agabian, N. (1977) Envelope-associated nucleoid from Caulobacter crescentus stalked and swarmer cells. *J Bacteriol* **132**: 294–301.

Goley, E.D., Dye, N.A., Werner, J.N., Gitai, Z., and Shapiro, L. (2010) Imaging-based identification of a critical regulator of FtsZ protofilament curvature in Caulobacter. *Mol Cell* **39**: 975–987.

Goley, E.D., Yeh, Y.-C., Hong, S.-H., Fero, M.J., Abeliuk, E., McAdams, H.H., and Shapiro, L. (2011) Assembly of the Caulobacter cell division machine. *Mol Microbiol* **80**: 1680–1698.

Roberts, R.C., Toochinda, C., Avedissian, M., Baldini, R.L., Gomes, S.L., and Shapiro, L. (1996) Identification of a Caulobacter crescentus operon encoding hrcA, involved in negatively regulating heat-inducible transcription, and the chaperone gene grpE. *J Bacteriol* **178**: 1829–1841.

Sundararajan, K., Miguel, A., Desmarais, S.M., Meier, E.L., Huang, K.C., and Goley, E.D. (2015) The bacterial tubulin FtsZ requires its intrinsically disordered linker to direct robust cell wall construction. *Nat Commun* **6**: 7281.

Thanbichler, M., Iniesta, A.A., and Shapiro, L. (2007) A comprehensive set of plasmids for vanillate- and xylose-inducible gene expression in Caulobacter crescentus. *Nucleic Acids Res* **35**: e137.

Thanbichler, M., and Shapiro, L. (2006) MipZ, a Spatial Regulator Coordinating Chromosome Segregation with Cell Division in Caulobacter. *Cell* **126**: 147–162.
